# MORC2 regulates RBM39-mediated CDK5RAP2 alternative splicing to promote EMT and metastasis in colon cancer

**DOI:** 10.1038/s41419-024-06908-y

**Published:** 2024-07-24

**Authors:** Yuxin He, Yangguang Shao, Zhihui Zhou, Tingting Li, Yunling Gao, Xue Liu, Gang Yuan, Gaoxiang Yang, Lili Zhang, Feng Li

**Affiliations:** Department of Cell Biology, Key Laboratory of Cell Biology, National Health Commission of the PRC and Key Laboratory of Medical Cell Biology, Ministry of Education of the PRC, School of Life Sciences, China Medical University, No. 77, Puhe Road, Shenyang North New Area, Shenyang, Liaoning 110122 China

**Keywords:** Epithelial-mesenchymal transition, Metastasis

## Abstract

Colorectal carcinogenesis and progression are associated with aberrant alternative splicing, yet its molecular mechanisms remain largely unexplored. Here, we find that Microrchidia family CW-type zinc finger 2 (MORC2) binds to RRM1 domain of RNA binding motif protein 39 (RBM39), and RBM39 interacts with site 1 of pre-CDK5RAP2 exon 32 via its UHM domain, resulting in a splicing switch of cyclin-dependent kinase 5 regulatory subunit associated protein 2 (CDK5RAP2) L to CDK5RAP2 S. CDK5RAP2 S promotes invasion of colorectal cancer cells in vitro and metastasis in vivo. Mechanistically, CDK5RAP2 S specifically recruits the PHD finger protein 8 to promote *Slug* transcription by removing repressive histone marks at the *Slug* promoter. Moreover, CDK5RAP2 S, but not CDK5RAP2 L, is essential for the promotion of epithelial-mesenchymal transition induced by MORC2 or RBM39. Importantly, high protein levels of MORC2, RBM39 and Slug are strongly associated with metastasis and poor clinical outcomes of colorectal cancer patients. Taken together, our findings uncover a novel mechanism by which MORC2 promotes colorectal cancer metastasis, through RBM39-mediated pre-CDK5RAP2 alternative splicing and highlight the MORC2/RBM39/CDK5RAP2 axis as a potential therapeutic target for colorectal cancer.

## Introduction

Colorectal cancer (CRC) is the third most diagnosed malignant disease and a major leading cause of cancer-related deaths in the world. In addition to gene mutations, aberrant pre-mRNA splicing becomes another major event that reflects the abnormalities of colorectal cancer cells [1]. Splicing factor-mediated alternative splicing (AS) has been demonstrated to be closely associated with colorectal cancer progression [2, 3]. For instance, SRSF6 regulates aberrant splicing of ZO-1 by directly binding to a motif in exon 23 of ZO-1, thereby promoting colorectal cancer progression [4]. Consequently, further investigation of alternative splicing events involved in the development and progression of colorectal cancer and their corresponding pathological features could contribute to the development of biomarkers and therapeutic targets for colorectal cancer.

Microrchidia family CW-type zinc finger 2 (MORC2) is a member of the highly conserved MORC family protein. It plays an important role in epigenetic gene silencing, DNA repair, lipogenesis and glucose metabolism [5–11]. MORC2 is found to be up-regulated in multiple cancers, including breast cancer, gastric cancer, colorectal cancer and liver cancer. It promotes the growth, migration, invasion and metastasis of cancer cells [9, 12–19]. However, the effect and mechanism of MORC2 on alternative splicing in colon cancer are not understood.

RNA binding motif protein 39 (RBM39) is an RNA-binding protein involved in transcriptional co-regulation and alternative splicing of pre-mRNAs [20, 21]. Several transcriptomic studies have also shown that RBM39 regulates alternative splicing of a variety of genes, such as genes related to the cell cycle [22–26]. RBM39-mediated alternative splicing is also involved in the carcinogenesis and progression of multiple cancers, including acute myeloid leukemia (AML), breast cancer, colorectal cancer and lung cancer [20, 21, 25, 27–29]. Indisulam (E7070), an anticancer sulfonamide, selectively recruits RBM39 to the CUL4-DCAF15 E3 ubiquitin ligase for proteasomal degradation and has good efficacy against neuroblastoma in mouse models [30]. However, whether MORC2 regulates RBM39-mediated alternative splicing in colon cancer is not known.

Cyclin-dependent kinase 5 regulatory subunit associated protein 2 (CDK5RAP2) is a pericentriolar matrix (PCM) protein that regulates centriole engagement and centrosome cohesion during the cell cycle. CDK5RAP2 organizes several PCM components, including pericentrin (PCNT) [31]. PCNT-CDK5RAP2 becomes essential for mitotic spindle formation when centrioles are absent [32]. CDK5RAP2 is involved in a variety of biological functions, including mitosis [33–37], cellular senescence [38–40], and transcriptional regulation [41, 42]. CDK5RAP2 is associated with several cancers, including breast cancer [43], oral squamous cell carcinoma [44] and haematological malignancies [45]. It has been found that there is a novel alternatively spliced variant form of CDK5RAP2, CDK5RAP2 S, which lacks exon 32 [46, 47]. However, the role and mechanism of CDK5RAP2 S in colorectal cancer remain elusive.

In this study, we identified a novel mechanism for MORC2 to promote CRC metastasis by manipulating the RBM39-mediated CDK5RAP2 splice isoform switch and epithelial-mesenchymal transition (EMT). In colorectal cancer, MORC2 binds to RBM39 to promote alternative splicing of pre-CDK5RAP2, converting CDK5RAP2 L into a variant CDK5RAP2 S. CDK5RAP2 S promotes invasion and metastasis of colon cancer cells in vitro and in vivo. These findings provide novel mechanistic insights into the role of MORC2 in promoting colorectal cancer metastasis and highlight the MORC2/RBM39/CDK5RAP2 axis as a potential therapeutic target for colorectal cancer.

## Results

### MORC2 directly interacts with RBM39

**Fig. 1:**
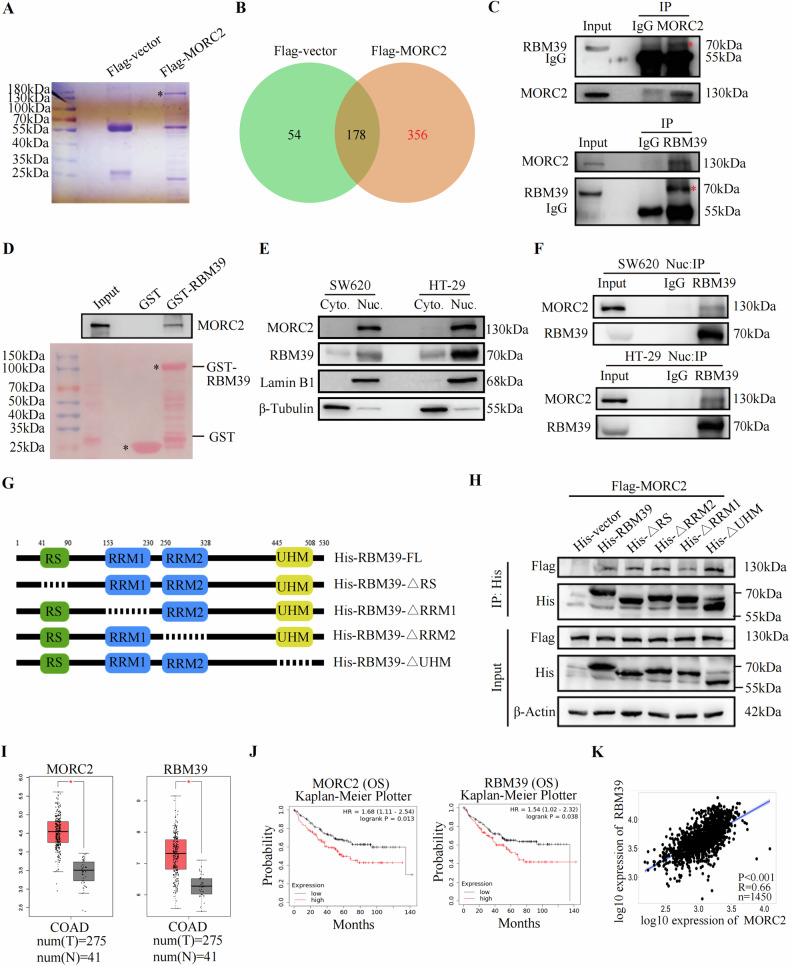
MORC2 directly interacts with RBM39 in colon cancer cells. **A** Lysates of HCT116 cells stably transfected with Flag or Flag-MORC2 were immunoprecipitated with anti-Flag antibody, followed by SDS-PAGE, coomassie brilliant blue staining and mass spectrum analysis (MS). **B** Venn diagram showed 356 proteins that specifically interact with Flag-MORC2. **C** Cell lysates were immunoprecipitated with the indicated antibody or IgG. Precipitates were analyzed by western blotting using the indicated antibodies. Red stars indicate RBM39 protein. **D** For GST pull-down assay, GST or GST-RBM39 fusion protein was incubated with Flag-MORC2 translated in vitro. Bound proteins were detected with western blotting. The lower figure shows Ponceau red staining. Black stars indicate GST and GST-RBM39 protein. **E** Nuclear (Nuc.) and cytoplasmic (Cyto.) fractions were isolated from SW620 or HT-29 cells. The expression of MORC2 and RBM39 was detected by western blot analysis. Lamin B1 and β-Tubulin served as positive controls for nuclei or cytoplasm proteins, respectively. **F** The nuclear proteins of SW620 or HT-29 cells were further subjected to immunoprecipitation with IgG or RBM39 antibodies, followed by blotting with MORC2 and RBM39 antibodies, respectively. **G** Diagrammatic representation of the full-length (His-RBM39-FL) and four domain-deleted His-tagged RBM39 plasmids (His-RBM39-ΔRS, His-RBM39-ΔRRM1, His-RBM39-ΔRRM2 and His-RBM39-ΔUHM). **H** His-vector, His-full-length and four domain-deleted RBM39 plasmids were transfected into HCT116 cells. Cell lysates were immunoprecipitated with His antibody. Precipitates were analyzed by western blotting using the indicated antibodies. **I** Box plots show MORC2 and RBM39 expression in colon adenocarcinoma tissues (T) and adjacent non-cancerous tissues (N) from the GEPIA database (http://gepia.cancer-pku.cn/). Red indicates colon adenocarcinoma tissues, and gray indicates adjacent non-cancerous tissues, *P < 0.05. **J** Relationship between MORC2 or RBM39 expression and the overall survival of colon cancer patients obtained from Kaplan-Meier Plotter database (https://kmplot.com/analysis/). **K** Correlation between MORC2 and RBM39 expression from TNMplot database (https://tnmplot.com/analysis/).

To further investigate the mechanism of MORC2 in the development of colorectal cancer, we used immunoprecipitation combined with mass spectrometry to identify proteins that interacted with MORC2. The analysis revealed 356 proteins that specifically bound to MORC2 (Fig. 1A, B). KOG analysis found MORC2 ranked first in relation to RNA processing and modification (Supplementary Fig. S1A). GO analysis further suggested that MORC2 participated in alternative splicing (Supplementary Fig. S1B). We ranked splicing factors that bind to MORC2 and selected RBM39 (Supplementary Fig. S1C), an RNA-binding protein that promoted the survival of colorectal cancer cells by regulating the splicing of various genes [20, 21, 27]. Co-IP experiments validated the interaction between endogenous MORC2 and RBM39 in SW480 cells (Fig. 1C). We also found that knockdown of MORC2 down-regulated RBM39 expression, suggesting that MORC2 promoted RBM39 expression (Supplementary Fig. S1D–G). Furthermore, using GST pull-down assays, we found that in vitro translated MORC2 bound GST-RBM39 directly (Fig. 1D). Our results further showed that MORC2 and RBM39 mainly localized in the nucleus and bound in the nucleus (Fig. 1E, F). RBM39 contains one RS domain, two canonical RRM domains (RRM1 and RRM2) and one non-canonical RRM domain, which is commonly known as the U2AF homology motif (UHM) (Fig. 1G) [21]. Further IP experiments showed that the deletion of the RRM1 domain in RBM39 reduced its interaction with MORC2, indicating that RBM39 interacts with MORC2 via the RRM1 domain of RBM39 (Fig. 1H). GEPIA database analysis showed high MORC2 and RBM39 mRNA expression in colon adenocarcinoma tissues (Fig. 1I). Patients with high MORC2 or RBM39 expression had a shorter overall survival (Fig. 1J). Importantly, MORC2 was positively correlated with RBM39 expression in colon cancer (Fig. 1K). Taken together, these data indicate the direct interaction between MORC2 and RBM39 in the nucleus of colon cancer cells.

### Alternative splicing of pre-CDK5RAP2 is regulated by both RBM39 and MORC2

**Fig. 2:**
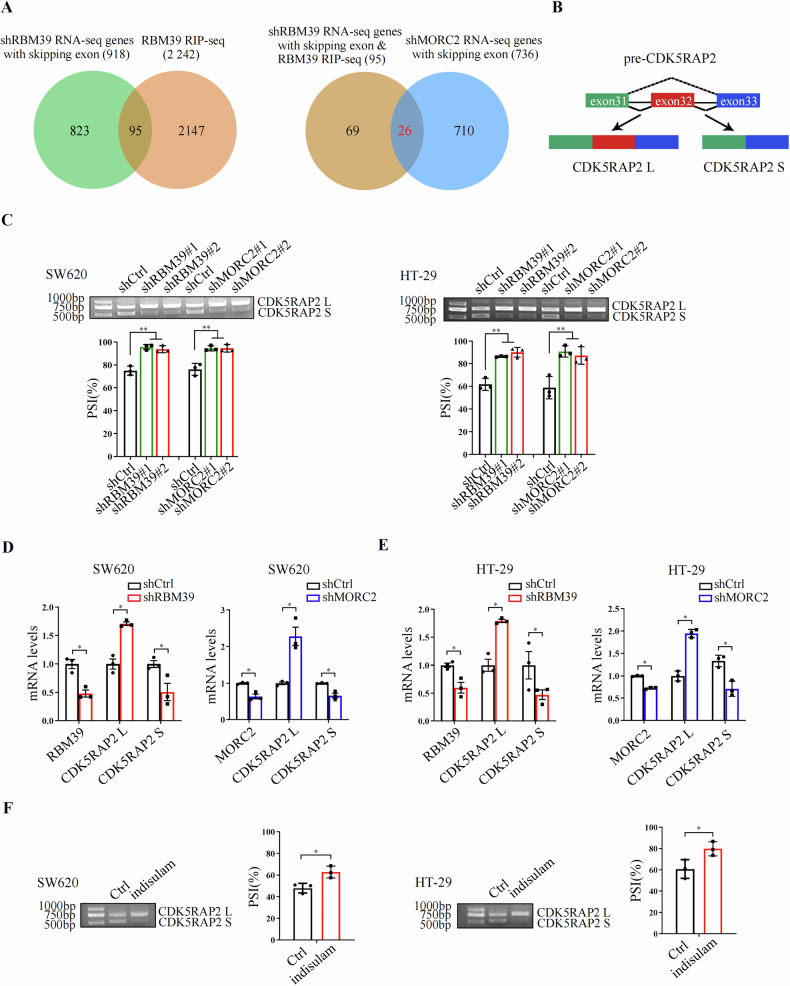
Alternative splicing of pre-CDK5RAP2 is regulated by both RBM39 and MORC2. **A** Venn diagram illustrates the overlap between genes by RBM39 RIP-seq and shRBM39 RNA-seq genes with exon-skipping (left panel). The right panel venn diagram shows the overlap between 95 genes and shMORC2 RNA-seq genes with skipping exon. **B** Schematic representation of pre-CDK5RAP2 alternative splicing. Exons are represented by green, red and blue rectangles, introns by black lines, and splicing pattern by dashed lines. **C** RT-PCR was used to detect the effects of RBM39 knockdown (shRBM39) and MORC2 knockdown (shMORC2) on CDK5RAP2 L and CDK5RAP2 S mRNA expression. The bottom panel shows the quantitative statistics of percent spliced in (PSI). *n* = 3, **P < 0.01. **D**, **E** RT-qPCR was used to detect the effect of RBM39 knockdown or MORC2 knockdown on CDK5RAP2 L and CDK5RAP2 S mRNA expression. *n* = 3, *P < 0.05. **F** RT-PCR was used to detect the effects of indisulam (0.125 μM, 8 h) on CDK5RAP2 L and CDK5RAP2 S mRNA expression. The right panel shows the quantitative statistics of percent spliced in (PSI). *n* = 3, *P < 0.05.

As a crucial splicing factor, RBM39 regulates the alternative splicing of pre-mRNA. Therefore, we first performed RNA-seq to reveal RBM39-regulated AS events (Supplementary Fig. S2A). Next, we performed RIP-seq to identify RBM39-associated transcriptome (Supplementary Fig. S2B). Considering that most splicing events were SEs (skipped exons, Supplementary Fig. S2A), we combined data from RIP-seq and RNA-seq (SE) and found 95 closely targeted events (Fig. 2A). To identify genes whose alternative splicing was regulated by both RBM39 and MORC2, we further performed shMORC2 RNA-seq (Supplementary Fig. S2C). Then we combined shMORC2 RNA-seq (SE) with the 95 RBM39 targeted SE events, and found 26 SE events (Fig. 2A, Supplementary Table 1, Supplementary Table 2). We selected several genes of interest, including CDK5RAP2, obscurin-like cytoskeletal adaptor 1 (OBSL1), microtubule affinity regulating kinase 2 (MARK2), zinc finger protein 35 (ZNF35) and FERM domain-containing 8 (FRMD8), and employed RT-qPCR and RT-PCR for experimental validation (Fig. 2B–E, Supplementary Fig. S2D–H). Interestingly, we found that the knockdown of RBM39 or MORC2 caused an isoform switch of CDK5RAP2 S (lacking exon 32) to CDK5RAP2 L (containing exon 32) (Fig. 2B–E). Meanwhile, indisulam, a molecular glue that promotes the degradation of RBM39, could also cause the isoform switch of CDK5RAP2 S to CDK5RAP2 L (Fig. 2F).

### MORC2 promotes RBM39-mediated alternative splicing of pre-CDK5RAP2

**Fig. 3:**
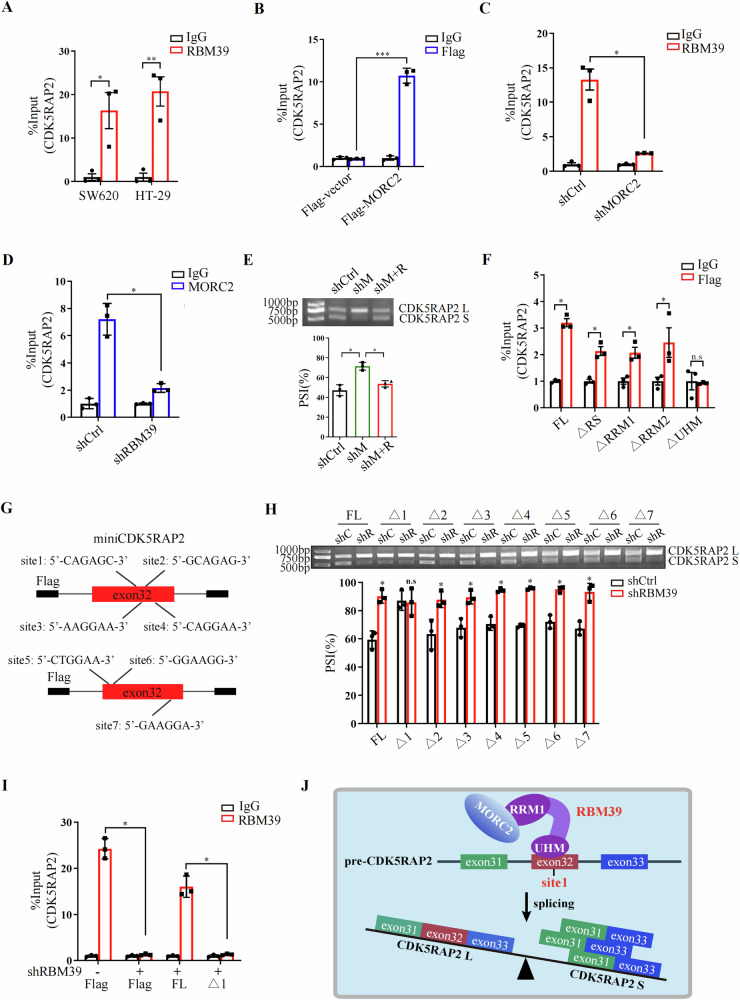
MORC2 promotes RBM39-mediated alternative splicing of pre-CDK5RAP2. **A**, **B** RIP was used to detect the binding of RBM39 (**A**) or MORC2 (**B**) to CDK5RAP2 pre-mRNA. IgG was used as a negative control, and the figure shows the quantitative statistics of RT-qPCR. *n* = 3, *P < 0.05, **P < 0.01, ***P < 0.001. **C**, **D** RIP assays were performed using control (shCtrl) and MORC2 knockdown (shMORC2) (**C**) or RBM39 knockdown (shRBM39) (**D**) SW620 cells to detect the binding of RBM39 (**C**) or MORC2 (**D**) to CDK5RAP2 pre-mRNA. IgG was used as a negative control, and the figure shows the quantitative statistics of RT-qPCR. *n* = 3, *P < 0.05. **E** RT-PCR was used to detect the effects of MORC2 knockdown (shM), MORC2 knockdown and RBM39 overexpression (shM+R) on CDK5RAP2 mRNA expression. The bottom panel shows the quantitative statistics of percent spliced in (PSI). *n* = 3, *P < 0.05. **F** Full length (FL) and four deletion Flag-tagged RBM39 plasmids were transfected into SW620 cells, and RIP was performed to determine the binding domain of RBM39 to CDK5RAP2 pre-mRNA. IgG served as a negative control. *n* = 3, *P < 0.05, n.s indicates no significance. **G** Model shows miniCDK5RAP2 with deletion of potential RBM39 binding sites (Δ1–Δ7). **H** RT-PCR was used to detect CDK5RAP2 L and CDK5RAP2 S mRNAs in control (shC) and RBM39 knockdown (shR) SW620 cells transfected with miniCDK5RAP2 plasmids with deletion of potential RBM39 binding sites (Δ1–Δ7). The bottom panel shows the quantitative statistics of percent spliced in (PSI). *n* = 3, *P < 0.05, n.s indicates no significance. **I** Control and RBM39 knockdown SW620 cells were transfected with Flag vector, miniCDK5RAP2-FL or miniCDK5RAP2-Δ1 plasmid, followed by RIP with anti-RBM39 or anti-IgG antibodies. IgG served as a negative control. *n* = 3, *P < 0.05. **J** Working model shows that MORC2 promotes RBM39-mediated alternative splicing of pre-CDK5RAP2.

To explore the mechanism by which MORC2 and RBM39 promoted the alternative splicing of pre-CDK5RAP2, we first verified that both RBM39 and MORC2 bound to pre-CDK5RAP2 using RIP-qPCR (Fig. 3A, B). Importantly, the knockdown of MORC2 inhibited the binding of RBM39 to pre-CDK5RAP2 (Fig. 3C), indicating that MORC2 was essential for RBM39-mediated alternative splicing of CDK5RAP2. Meanwhile, knockdown of RBM39 inhibited the binding of MORC2 to pre-CDK5RAP2 (Fig. 3D). Overexpression of RBM39 abolished the isoform switch of CDK5RAP2 S to CDK5RAP2 L caused by MORC2 knockdown (Fig. 3E), illustrating that RBM39 is necessary for MORC2-mediated alternative splicing of CDK5RAP2. Moreover, an RIP assay expressing differentially truncated Flag-tagged RBM39 protein (ΔRS, ΔRRM1, ΔRRM2 and ΔUHM) revealed that the UHM domain of RBM39 is responsible for its binding to pre-CDK5RAP2 (Fig. 3F). To further examine the binding site of RBM39 on pre-CDK5RAP2, we generated miniCDK5RAP2 plasmids with individual deletion of seven potential RBM39 binding sites on pre-CDK5RAP2 (Fig. 3G). RT-PCR analysis of RBM39 knockdown cells and control cells transfected with these miniCDK5RAP2 vectors showed that deletion of site 1 abolished the isoform switch of CDK5RAP2 S to CDK5RAP2 L caused by RBM39 knockdown (Fig. 3H). RIP experiments also indicated that RBM39 binds to site 1 of pre-CDK5RAP2 (Fig. 3I). Taken together, these data demonstrate that MORC2 interacts with the RRM1 domain of RBM39, and RBM39 binds to site 1 of pre-CDK5RAP2 exon 32, promoting exon 32 skipped splicing and the generation of the CDK5RAP2 S splicing variant (Fig. 3J).

### CDK5RAP2 S promotes migration and invasion of colon cancer cells in vitro and metastasis in vivo

**Fig. 4:**
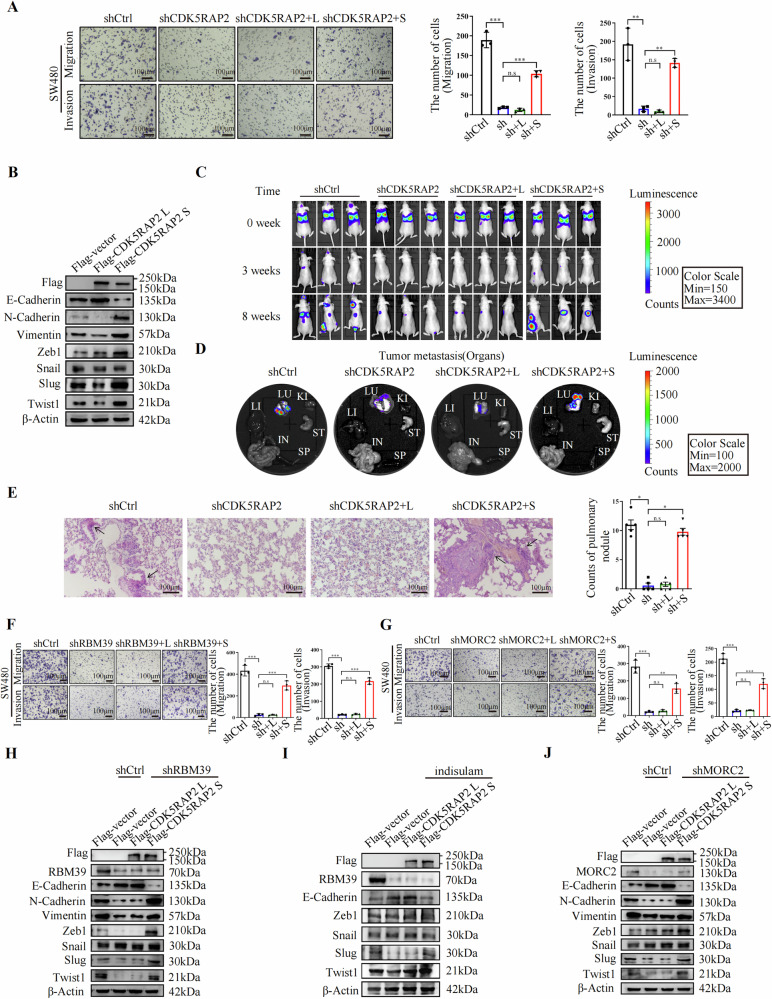
MORC2 and RBM39 promote EMT, migration and invasion of colon cancer cells via CDK5RAP2 S. **A** Migratory and invasive capacities of the cells were evaluated by transwell assays or matrigel-coated transwell assays. Representative photomicrographs of transwell results were taken under ×100 original magnifications. Scale bar, 100 μm. The right panel shows the statistical results of cell numbers. *n* = 3, **P < 0.01, ***P < 0.001, n.s indicates no significance. **B** SW620 cells were transfected with Flag-vector, Flag-CDK5RAP2 L or Flag-CDK5RAP2 S followed by western blot analysis to detect the indicated proteins. **C** Representative BLI images of three animals at the indicated time points after tail vein injection of SW480-Luc stably transfected cells (five mice per group). **D** Bioluminescence images of selected mouse organs at the 8th week after injection. Abbreviations: LU lung, KI kidney, ST stomach, SP spleen, IN intestine, LI liver. **E** Representative images of HE staining of lung tissues from mice. Original magnification, ×200. Scale bar, 100 μm. The right panel shows the statistical results of the number of pulmonary nodules, *n* = 5, *P < 0.05, n.s indicates no significance. **F**, **G** Migratory and invasive capacities of SW480-shCtrl, SW480-shRBM39, SW480-shRBM39+L (CDK5RAP2 L) and SW480-shRBM39+S (CDK5RAP2 S) cells (**F**) or SW480-shCtrl, SW480-shMORC2, SW480-shMORC2+L (CDK5RAP2 L) and SW480-shMORC2+S (CDK5RAP2 S) cells (**G**) were measured by transwell assays or matrigel-coated transwell assays. Representative photomicrographs of transwell results were taken under ×100 original magnifications. Scale bar, 100 μm. The right panel shows the statistical results of cell numbers. *n* = 3, **P < 0.01, ***P < 0.001, n.s indicates no significance. **H** The indicated protein levels were measured by western blot analysis in SW620 cells with co-transfection of shRBM39 and Flag-CDK5RAP2 L or Flag-CDK5RAP2 S. **I** Western blot analysis of the indicated protein expression in SW620 cells transfected with Flag-vector, Flag-CDK5RAP2 L and Flag-CDK5RAP2 S treated by indisulam or not. **J** Western blot analysis of the indicated protein expression in SW620 cells with co-transfection of shMORC2 and Flag-CDK5RAP2 L or Flag-CDK5RAP2 S.

Next, we investigated the molecular function of the two CDK5RAP2 isoforms in colon cancer cells. By overexpression of CDK5RAP2 L or CDK5RAP2 S after CDK5RAP2 knockdown via lentiviral infection in SW480-luc cells (Supplementary Fig. S3A, B), we found that CDK5RAP2 S, but not CDK5RAP2 L promoted the migration and invasion of colon cancer cells (Fig. 4A). Then the expression of EMT markers and several important EMT transcription factors were examined by western blotting. The results showed that CDK5RAP2 L overexpression up-regulated E-cadherin and down-regulated N-cadherin, Vimentin, Slug, and Twist1 expression. Whereas overexpression of CDK5RAP2 S down-regulated E-cadherin and up-regulated N-cadherin, Vimentin, Zeb1, Slug and Twist1 expression (Fig. 4B), indicating that CDK5RAP2 L and CDK5RAP2 S might have different roles in EMT of colon cancer cells. Moreover, the four groups of SW480-luc cells were then injected into the tail vein of nude mice to detect the effect of CDK5RAP2 L or CDK5RAP2 S on tumor metastasis using live animal bioluminescence imaging (BLI). At 10 min after the injection of the cancer cells, equal levels of bioluminescence were observed in the mouse lungs (Fig. 4C, top row). The mice were imaged once a week, with the final images taken immediately prior to sacrifice. The results showed that metastasis began to occur in the third week after injection in control and shCDK5RAP2 + CDK5RAP2 S groups, while no metastasis was found in shCDK5RAP2 and shCDK5RAP2 + CDK5RAP2 L groups at this time (Fig. 4C, middle row). At the study endpoint, shCDK5RAP2 and shCDK5RAP2 + CDK5RAP2 L groups exhibited significantly less lung metastasis compared with the control group, while shCDK5RAP2 + CDK5RAP2 S groups showed markedly more lung metastasis (Fig. 4C, bottom row). The fluorescence imaging of lung tissues and the HE staining results of lung tissues presented consistent results (Fig. 4D, E). Taken together, these findings demonstrate that CDK5RAP2 S promotes metastasis of colon cancer cells in vivo.

### MORC2 and RBM39 promote EMT, migration and invasion of colon cancer cells via CDK5RAP2 S

MORC2 has been shown to promote metastasis of triple-negative breast cancer and cholangiocarcinoma by regulating the EMT process [18, 19]. However, the role of RBM39 in the EMT process is unclear. Then we verified that MORC2 and RBM39 promoted EMT in colon cancer cells (Supplementary Fig. S3C). Moreover, our data showed that CDK5RAP2 S overexpression, but not CDK5RAP2 L, partially rescued the inhibitory effect of migration and invasion induced by RBM39 knockdown or MORC2 knockdown (Fig. 4F, G). Furthermore, we found that overexpression of CDK5RAP2 S, but not CDK5RAP2 L, reversed the promoting effect of RBM39 knockdown on E-cadherin expression and the inhibitory effect on N-cadherin, Vimentin, Zeb1, Slug and Twist1 expression (Fig. 4H), and a consistent result was obtained using indisulam (Fig. 4I, Supplementary Fig. S3D). In addition, a similar result was obtained when MORC2 was knocked down (Fig. 4J). Taken together, MORC2 and RBM39 promote EMT, migration and invasion of colon cancer cells via CDK5RAP2 S.

### PHF8 promotes EMT in colon cancer cells

**Fig. 5:**
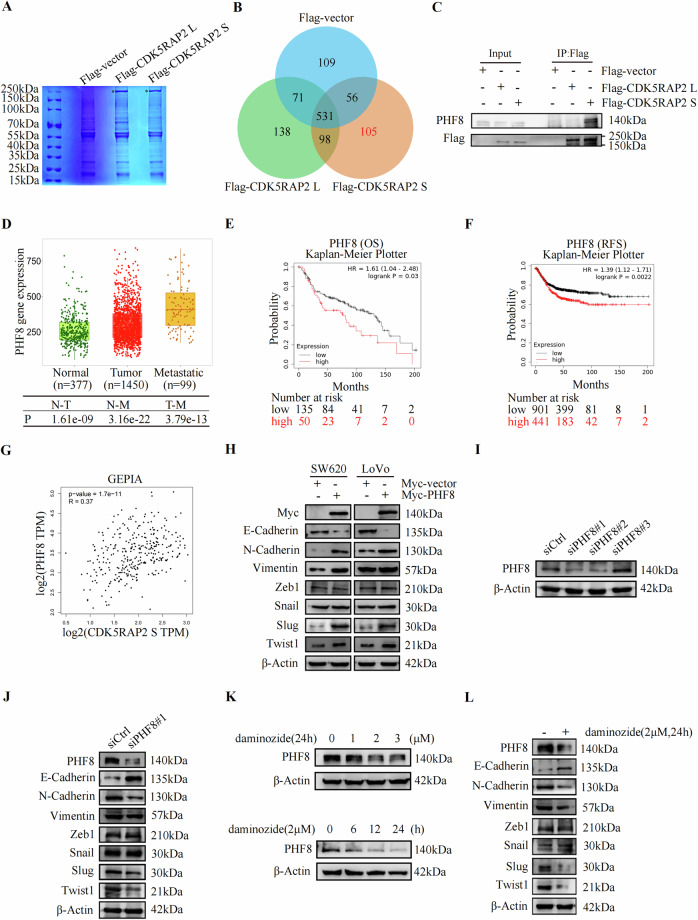
PHF8 promotes EMT in colon cancer cells. **A** Lysates of SW620 cells transfected with Flag, Flag-CDK5RAP2 L or Flag-CDK5RAP2 S were immunoprecipitated with anti-Flag antibody, followed by SDS-PAGE, coomassie brilliant blue staining and mass spectrum (MS) analysis. **B** Venn diagram shows proteins that specifically interact with CDK5RAP2 L or CDK5RAP2 S. **C** SW620 cells were transfected with Flag-vector, Flag-CDK5RAP2 L or Flag-CDK5RAP2 S, and the lysates were immunoprecipitated with anti-Flag antibody. Precipitates were analyzed by western blotting using anti-Flag or anti-PHF8 antibodies. **D** Box plot shows *PHF8* expression in adjacent non-cancerous tissues (N, Normal), colon cancer tissues (T, Tumor) and metastatic colon cancer tissues (M, Metastatic) from TNMplot database. **E**, **F** Relationship between *PHF8* expression and the overall survival (E) or recurrence-free survival (F) of colon cancer patients obtained from Kaplan-Meier Plotter database. **G** The correlation between *CDK5RAP2 S* and *PHF8* expression from the GEPIA database. **H** Western blot analysis of the indicated protein expression in SW620 or LoVo cells transfected with Myc-vector or Myc-PHF8. **I** SW620 cells were transfected with scramble siRNA or siRNAs targeting PHF8. The relative level of PHF8 protein was determined by western blot analysis. **J** Western blot analysis of the indicated protein expression in SW620 cells transfected with scramble siRNA or siPHF8#1. **K** Western blot analysis of PHF8 expression in SW620 cells treated with different concentrations of daminozide for 24 h (upper panel) and 2 μM daminozide for different times (bottom panel). **L** Western blot analysis of the indicated protein expression in SW620 cells treated with daminozide (2 μM, 24 h).

To investigate the mechanism by which CDK5RAP2 S promotes EMT, we used immunoprecipitation combined with mass spectrometry to screen for proteins that bind to CDK5RAP2 L or CDK5RAP2 S (Fig. 5A). 105 proteins were identified that specifically bound to CDK5RAP2 S, including histone demethylase PHD finger protein 8 (PHF8) (Supplementary Figs. S4, 5B). Furthermore, Co-IP experiments showed that exogenous CDK5RAP2 S, but not CDK5RAP2 L bound to endogenous PHF8 in SW620 cells (Fig. 5C). TNMplot database analysis showed that PHF8 was up-regulated in colon cancer (Tumor) and the expression of PHF8 was higher in metastatic cancer (Metastatic) than in primary cancer (Tumor) (Fig. 5D). Moreover, Kaplan-Meier Plotter database analysis showed that patients with higher PHF8 expression had a shorter overall survival and recurrence-free survival (Fig. 5E, F). Importantly, CDK5RAP2 S expression level was positively correlated with PHF8 expression level in the GEPIA database (Fig. 5G). Western blotting result revealed that PHF8 overexpression down-regulated E-cadherin and up-regulated N-cadherin and vimentin, as well as Slug and Twist1 expression (Fig. 5H). In contrast, PHF8 knockdown by siRNAs led to the opposite result (Fig. 5I, J). Next, by using daminozide, an inhibitor of PHF8 [48], we observed consistent result with that of silencing PHF8 (Fig. 5K, L). Collectively, these data indicate that CDK5RAP2 S specifically interacts with PHF8, and that PHF8 may promote EMT in colon cancer cells.

### CDK5RAP2 S recruits PHF8 to promote *Slug* transcription

**Fig. 6:**
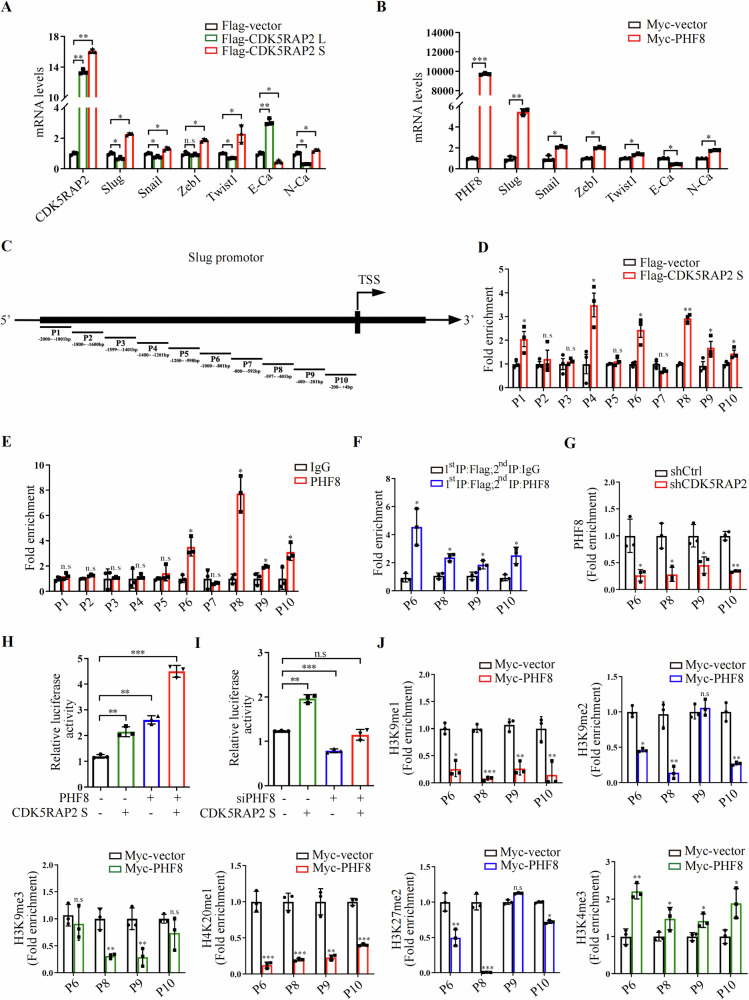
CDK5RAP2 S recruits PHF8 to promote *Slug* transcription. **A**, **B** SW620 cells were transfected with Flag-vector, Flag-CDK5RAP2 L or Flag-CDK5RAP2 S (**A)**, Myc-vector, Myc-PHF8 (**B**), followed by RT-qPCR to detect the mRNA level of the indicated genes. *n* = 3, *P < 0.05, **P < 0.01, ***P < 0.001, n.s indicates no significance. **C** Schematic diagram of primers for *Slug* promoter sequences. 10 primer sets with a 200 bp partition were designed for ChIP-qPCR. **D** SW620 cells were transfected with Flag-vector or Flag-CDK5RAP2 S, followed by ChIP assays with anti-flag antibodies. The P1–P10 fragment of the *Slug* promoter was amplified by qPCR. *n* = 3, *P < 0.05, **P < 0.01, n.s indicates no significance. **E** SW620 cells were subjected to ChIP assays with antibodies as indicated, followed by qPCR with primers amplifying the *Slug* promoter P1–P10 fragments. *n* = 3, *P < 0.05, n.s indicates no significance. **F** Flag-CDK5RAP2 S plasmid was transfected into SW620 cells. Then ChIP Re-ChIP assay was performed. Soluble chromatin was first immunoprecipitated with flag antibody (1st IP). The complexes eluted from the first IP were equally divided into two aliquots, followed by re-immunoprecipitation with IgG or PHF8 antibodies (2nd IP), respectively. The P6, P8, P9, and P10 fragments of the *Slug* promoter were amplified by qPCR. *n* = 3, *P < 0.05. **G** ChIP assays were performed using control (shCtrl) and CDK5RAP2 knockdown (shCDK5RAP2) SW620 cells with anti-PHF8 antibodies. The P6, P8, P9, and P10 fragment of the *Slug* promoter was amplified by qPCR. *n* = 3, *P < 0.05. **H**, **I** Luciferase reporter gene plasmid containing the *Slug* promoter, PHF8 and CDK5RAP2 S (H), siPHF8 and CDK5RAP2 S (I) were transfected into SW620 cells as indicated, and the *Slug* promoter activity was estimated by luciferase assays. *n* = 3, **P < 0.01, ***P < 0.001, n.s indicates no significance. **J** SW620 cells were transfected with Myc-vector and Myc-PHF8, and ChIP assays were performed with H3K9me1, H3K9me2, H3K9me3, H4K20me1, H3K27me2, and H3K4me3 antibodies. P6, P8, P9, and P10 fragments of the *Slug* promoter were amplified by qPCR. *n* = 3, *P < 0.05, **P < 0.01, ***P < 0.001, n.s indicates no significance.

To investigate the mechanism by which CDK5RAP2 S and PHF8 promote EMT in colon cancer, we performed RT-qPCR experiments. The results showed that CDK5RAP2 S or PHF8 overexpression up-regulated mRNA levels of *Slug*, *Snail, Zeb1, Twist1, N-cadherin*, and down-regulated *E-cadherin* (Fig. 6A, B). Among them, *Slug* was most significantly regulated by CDK5RAP2 S and PHF8. Next, we designed 10 primer sets for PCR after chromatin immunoprecipitation (ChIP) to confirm the binding of CDK5RAP2 S and PHF8 to the *Slug* promoter (Fig. 6C). As shown in Fig. 6D, the binding of CDK5RAP2 S was detected at the *Slug* promoter regions P1, P4, P6, P8, P9, and P10. Meanwhile, endogenous PHF8 could bind to the P6, P8, P9, and P10 regions of the *Slug* promoter (Fig. 6E). Importantly, ChIP Re-IP assays revealed that CDK5RAP2 S and PHF8 acted in a combinatorial fashion on the P6, P8, P9, and P10 regions of the *Slug* promoter (Fig. 6F). In addition, knockdown of CDK5RAP2 decreased the binding of PHF8 to *Slug* promoter (Fig. 6G). To provide additional evidence, luciferase dual reporter assays were performed, and the results showed that CDK5RAP2 S promoted *Slug* promoter activity independently and cumulatively with PHF8 (Fig. 6H). Moreover, silencing PHF8 suppressed the activity of the *Slug* promoter, which was partially reversed by CDK5RAP2 S co-transfection (Fig. 6I).

As PHF8 is a histone demethylase that activates transcription by removing repressive histone methylation markers [49–51], we wondered whether PHF8 up-regulated *Slug* transcription by its histone demethylase function. Interestingly, ChIP assays revealed that PHF8 overexpression reduced the different histone methylation marker levels at different degrees, H3K9me1 and H4K20me1 in the P6, P8, P9 and P10 regions, H3K9me2 and H3K27me2 in the P6, P8 and P10 regions, H3K9me3 in the P8 and P9 regions, while PHF8 overexpression stabilized H3K4me3 level in the P6, P8, P9 and P10 regions of the *Slug* promoter (Fig. 6J). Taken together, these data suggest that CDK5RAP2 S recruits PHF8 to the *Slug* promoter, and PHF8 exerts its histone demethylase function, thereby promoting *Slug* transcription.

### High protein levels of MORC2, RBM39 and Slug are associated with metastasis in colorectal cancer

**Fig. 7:**
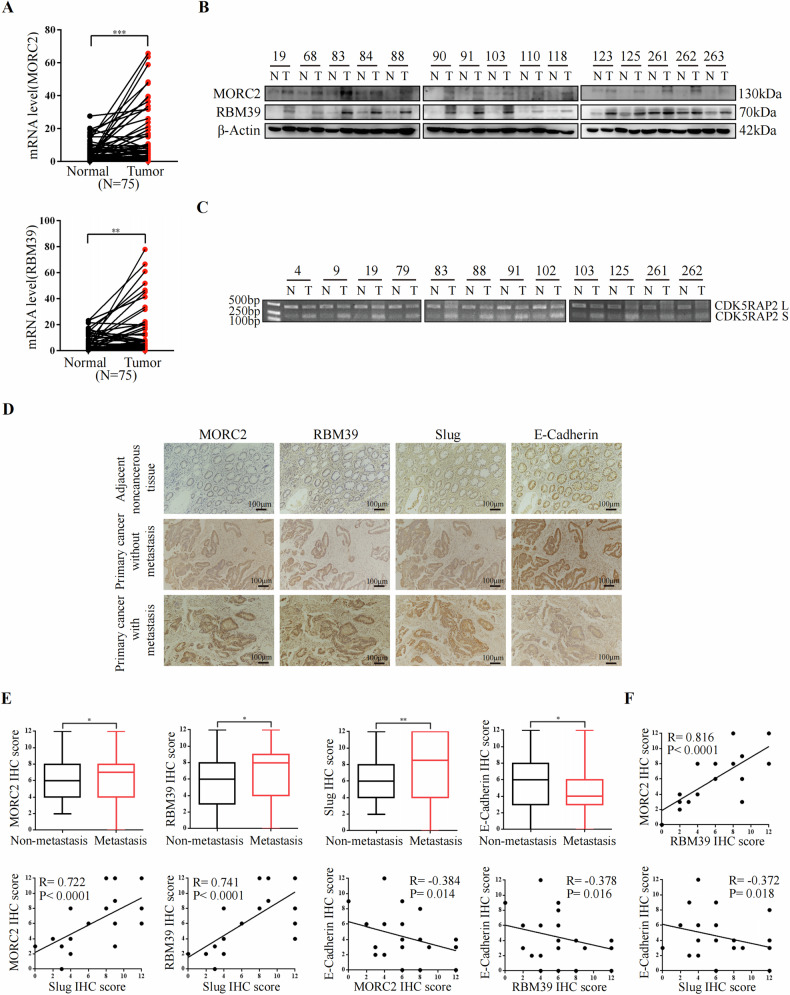
High protein levels of MORC2, RBM39 and Slug are associated with metastasis in colorectal cancer. **A**, **B** mRNA (**A**) and protein (**B**) expression of MORC2 and RBM39 in 75 pairs of clinical colorectal cancer tissues and adjacent non-cancerous tissues were detected by RT-qPCR (**A**) and western blot analysis (**B**). The representative 15 pairs were shown on B. N, matched adjacent non-cancerous tissues. T, tumor tissues. **P < 0.01, ***P < 0.001. **C** RT-PCR was used to detect the expression of CDK5RAP2 L and CDK5RAP2 S in paired colorectal cancer tissues. N, matched adjacent non-cancerous tissues. T, tumor tissues. **D** Representative immunohistochemical staining images of serial sections of adjacent non-cancerous tissues (*n* = 30), primary cancer without metastasis tissues (*n* = 60) and primary cancer with metastasis tissues (*n* = 40). Original magnification, ×100. Scale bar, 100 μm. Intensity value was expressed as immunohistochemistry score (IHC score). **E** IHC score analysis of MORC2, RBM39, Slug and E-cadherin expression in primary colorectal cancer tissues without metastasis (non-metastasis) (*n* = 60) and primary colorectal cancer tissues with metastasis (metastasis) (*n* = 40). *P < 0.05, **P < 0.01. **F** Pearson correlation analysis was used to analyze the correlation between MORC2 and RBM39, MORC2 and Slug, RBM39 and Slug, E-cadherin and MORC2, E-cadherin and RBM39, E-cadherin and Slug expression in colorectal cancer tissues.

Next, we analyzed the expression of MORC2 and RBM39 in patients with colorectal cancer. We found both RNA and protein expression levels of MORC2 and RBM39 were significantly higher in colorectal cancer tissues in our cohort (Fig. 7A, B). As shown in Table 1, MORC2 expression was significantly correlated with tumor size (*p* = 0.018), clinical stage (*p* = 0.003), lymph node metastasis (pN) (*p* = 0.010) and distant metastasis (pM) (*p* = 0.030). Meanwhile, the expression of RBM39 was significantly associated with the clinical stage (*p* = 0.046) (Table 2). We also found decreased expression of CDK5RAP2 L and increased expression of CDK5RAP2 S in colorectal cancer tissues (Fig. 7C). Finally, we performed immunohistochemical staining for serial sections of adjacent non-cancerous tissues (*n* = 30), primary cancer without metastasis tissues (*n* = 60), primary cancer with lymph node and distant metastasis tissues (*n* = 40). The results demonstrated that MORC2, RBM39, and Slug were highly expressed in colorectal cancer tissues with metastasis, while E-cadherin was not or lowly expressed (Fig. 7D, E). Correlation analysis revealed that MORC2 and RBM39, MORC2 and Slug, RBM39 and Slug expression were positively correlated in colorectal cancer with metastasis. Meanwhile, MORC2 and E-cadherin, RBM39 and E-cadherin, Slug and E-cadherin expression were negatively correlated (Fig. 7F).

**Table 1:** Correlation between MORC2 expression and clinicopathological characteristics of colorectal cancer patients.

Feature	*n*	MORC2 expression		*P*-value
		Lower	Higher	
*Age (y)*				
<65	34	14	20	0.386
≥65	41	21	20	
*Gender*				
Male	43	17	26	0.151
Female	32	18	14	
*Tumor size (cm)*				
<5	47	17	30	0.018*
≥5	28	18	10	
*Clinical stages*				
Stages I, II	42	26	16	0.003**
Stages III, IV	33	9	24	
*Depth of invasion (pT)*				
T1, T2	24	10	14	0.552
T3, T4	51	25	26	
*Lymph node metastasis (pN)*				
N0	44	26	18	0.010*
N1,N2	31	9	22	
*Distant metastasis (pM)*				
M0	70	35	35	0.030*
M1	5	0	5	
*Differentiation*				
Poorly, moderately	61	26	35	0.143
Highly	14	9	5	

χ^2^ test was used to analyze the association between MORC2 expression and clinical features

*P* values < 0.05 representing statistical significance are in bold. Specifically, *P < 0.05, **P < 0.01.

**Table 2:** Correlation between RBM39 expression and clinicopathological characteristics of colorectal cancer patients.

Feature	n	RBM39 expression		*P*-value
		Lower	Higher	
*Age (y)*				
<65	34	14	20	0.850
≥65	41	16	25	
*Gender*				
Male	43	18	25	0.703
Female	32	12	20	
*Tumor size (cm)*				
<5	47	18	29	0.697
≥5	28	12	16	
*Clinical stages*				
Stages I, II	42	21	21	0.046*
Stages III, IV	33	9	24	
*Depth of invasion (pT)*				
T1, T2	24	10	14	0.840
T3, T4	51	20	31	
*Lymph node metastasis (pN)*				
N0	44	21	23	0.104
N1, N2	31	9	22	
*Distant metastasis (pM)*				
M0	70	28	42	1.000
M1	5	2	3	
*Differentiation*				
Poorly, moderately	61	23	38	0.397
Highly	14	7	7	

*χ*^*2*^ test was used to analyze the association between RBM39 expression and clinical features

*P* values < 0.05 representing statistical significance are in bold. Specifically, *P < 0.05.

## Discussion

It is well documented that MORC2 inhibits the transcription of several target genes, such as CAIX, ArgBP2, p21^Waf/Cip1^, and thus promotes cancer progression [5, 13, 15]. Other studies showed that MORC2 was essential in DNA damage repair and lipogenesis [6, 7]. However, only one study found that MORC2 mutant M276I regulated hnRNPM-mediated CD44 splicing switch to promote invasion and metastasis in triple-negative breast cancer cells [18]. The role of MORC2 wild type in alternative splicing remains unclear. In this study, our data demonstrate the functional links between MORC2, RBM39 and the downstream CDK5RAP2 whose alternative splicing they regulate in EMT and metastasis of colon cancer cells.

RBM39 is a bifunctional RBP involved in transcriptional co-regulation and RNA splicing [20, 21]. Through the two functions, RBM39 participates in cell metabolism, cell cycle, cellular response to hypoxia and angiogenesis, thus plays an important role in cancer cell survival and cancer progression [25–29]. Indisulam-mediated RBM39 degradation causes splicing defects in a broad range of genes, including TRIM27, and inhibits proliferation of colon cancer cells [27]. Here, we identified a novel target gene CDK5RAP2, whose alternative splicing was regulated by RBM39 and MORC2, contributing to a better understanding of the mechanisms of RBM39 and MORC2 in colon cancer.

Except for RBM39, at least three E3 ligases (TRIM21, TRIM25, TRIP12) were discovered by mass spectrometry experiments to identify MORC2-binding proteins. Whether MORC2 protein can be degraded by ubiquitination has not been reported, so it is necessary to further explore whether MORC2 interacted with these three E3 ligases and can be degraded as a substrate for them. TRIM25 was a known E3 ubiquitin ligase of RBM39 [52], whether RBM39 also interacted with TRIM21 or TRIP12 remains to be verified. It is reported that TRIM25 promoted the ubiquitination of the splicing factor NONO and thus suppressed the splicing function of NONO [53]. Whether these three E3 ligases inhibit RBM39-mediated alternative splicing by promoting the ubiquitination of RBM39 remains to be verified. In addition, we found that MORC2 up-regulated the protein level of RBM39 (Supplementary Fig. S1D, E). It is very likely that MORC2 prevents RBM39 from the ubiquitination degradation by impeding the interaction between RBM39 and these three E3 ligases, especially TRIM25, thus enhancing the protein stability of RBM39, and therefore promoting the splicing function of RBM39. This needs to be further studied.

Alternative pre-mRNA splicing is often cooperatively regulated by multiple splicing factors. For instance, simultaneous binding of RBM39, U2AF65, PUF60 and SPF45 to SF3B1 has been reported [21]. Similarly, the DHX9-NONO-SFPQ complex regulates the oncogenic splicing switch of BIN1 in hepatocellular carcinoma [54]. Further study is needed to determine whether other partners have participated in the regulation of pre-CDK5RAP2 splicing by RBM39 and MORC2. In addition to discovering that MORC2 interacts with RBM39, we also found that MORC2 up-regulated the mRNA and protein levels of RBM39 (Supplementary Fig. S1D–G). Because MORC2 promotes development of an aggressive colorectal cancer phenotype [16] and RBM39 increases the survival rate and anchoring-independent growth of colorectal cancer cells [55], further study on the mechanism of RBM39 upregulation by MORC2 would be vital for therapy of patients with colorectal cancer.

It has been found that there is a novel alternatively spliced variant form of CDK5RAP2, CDK5RAP2 S, which lacks 237 nucleotide residues in exon 32 [46]. However, the role and mechanism of CDK5RAP2 S in colorectal cancer progression remain unclear. Here, our results indicate that CDK5RAP2 S regulated by MORC2 and RBM39 promotes colorectal cancer progression. It has been reported that CDK5RAP2 also localizes in the nucleus, where it acts as a transcriptional activator for *CENP-A* [41]. CDK5RAP2 serves to maintain centromeric chromatin integrity partly through CENP-A [41]. Moreover, CDK5RAP2 is required for spindle checkpoint function by positively regulating the promoters of *BUBR1* and *MAD2* [42]. These studies suggest that CDK5RAP2 functions as a transcriptional activator. However, the role of CDK5RAP2 S in transcriptional regulation is unknown. Here, we illustrated that CDK5RAP2 S interacts with PHF8 to activate *Slug* transcription. Previous evidence demonstrated that PHF8 contributes to EMT and promotes malignant progression and metastasis in breast cancer, prostate cancer and liver cancer. [49, 56–58]. In accordance, our data showed that PHF8 was overexpressed and associated with poor prognosis in colon cancer. As a histone demethylase, PHF8 activates various genes by erasing repressive histone markers, including H3K9me1/2, H3K27me2 and H4K20me1 [49]. Consistently, our results demonstrated that PHF8 up-regulated *Slug* transcription by removing repressive histone methylation markers.

**Fig. 8:**
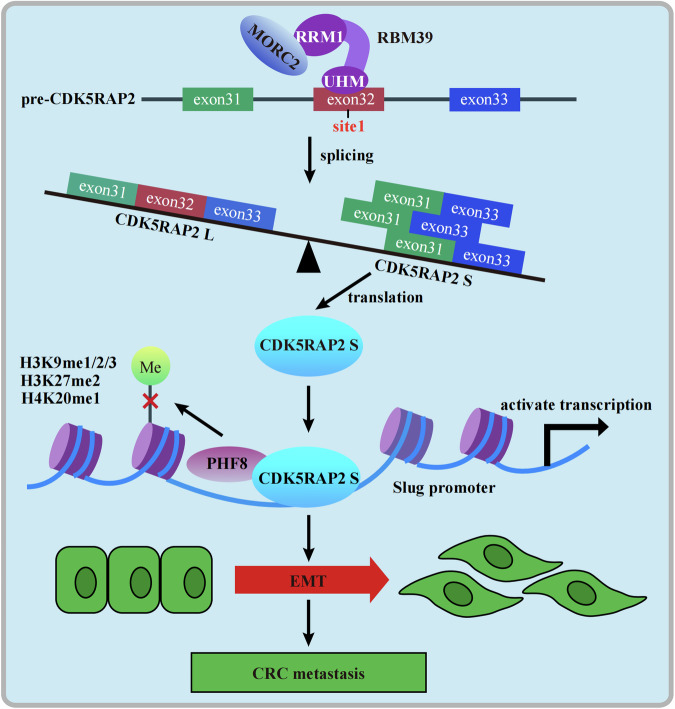
MORC2 promotes EMT and metastasis of colorectal cancer via RBM39-mediated alternative splicing. The working model indicates that MORC2 binds to the RRM1 domain of RBM39, and that RBM39 interacts with site 1 of pre-CDK5RAP2 exon 32 via its UHM domain, promoting the production of the CDK5RAP2 S splicing isoform. CDK5RAP2 S protein recruits PHF8 to activate *Slug* transcription by removing repressive histone markers (H3K9me1/2/3, H3K27me2, and H4K20me1) at the *Slug* promoter, thereby promoting EMT and metastasis of colorectal cancer cells.

In summary, our results reveal a novel mechanism by which MORC2 promotes colorectal cancer development by promoting the isoform switch of CDK5RAP2 L to CDK5RAP2 S through interacting with RBM39 (Fig. 8). Our findings not only provide a novel mechanism but also suggest a treatment strategy of targeting the MORC2/RBM39/CDK5RAP2 axis for colorectal cancer therapy.

## Materials and methods

### Cell culture

HCT116, SW480, SW620, HT-29 and LoVo cells were obtained from the Shanghai cell bank of Chinese Academy of Sciences. All cell lines were authenticated and were verified to be mycoplasma negative. Cells were cultured in RPMI-1640 medium (Gibco, USA), supplemented with 10% FBS, 1% penicillin/streptomycin and 1% glutamine at 37 °C in 5% CO_2_ and 95% air. Large frozen stocks were made for future use. All cell lines were used within 15 passages (less than 2 months) after reviving from the frozen stocks. Trypan blue exclusion assays were used to assess cell growth and survival.

### Plasmid construction and cell transfection

His-RBM39, Flag-CDK5RAP2 L, Flag-CDK5RAP2 S and Myc-PHF8 expression plasmids were purchased from Sino Biological Inc. (Beijing, China). His-RBM39-ΔRS, His-RBM39-ΔRRM1, His-RBM39-ΔRRM2, and His-RBM39-ΔUHM were purchased from Sangon Biotech (Shanghai, China). Minigene of CDK5RAP2 was constructed by BGI (Beijing, China). GST-tagged RBM39 was constructed by PCR and subcloned into pGEX-4T-2 vector (Amersham Biosciences, USA); and Flag-tagged RBM39-Full Length and a series of RBM39 domain deletion mutants (Flag-RBM39-ΔRS, Flag-RBM39-ΔRRM1, Flag-RBM39-ΔRRM2, Flag-RBM39-ΔUHM) were constructed by PCR and subcloned into pcDNA3.1-Flag vector (Invitrogen, USA). The Slug promoter reporter plasmid was a gift from Ceshi Chen (Kunming Institute of Zoology, Chinese Academy of Sciences, China). Details of the plasmids used are given in Supplementary Table 3 and details of the primers used are given in Supplementary Table 4. Transient transfection was carried out using Lipofectamine 3000 (Invitrogen, Carlsbad, CA, USA) according to the manufacturer’s instructions.

### Lentivirus and siRNA infection

Lentiviruses harboring Flag-vector, Flag-MORC2, nonsilencing-shRNA(shCtrl), shMORC2, shRBM39, Flag-CDK5RAP2 L, Flag-CDK5RAP2 S and shCDK5RAP2 were purchased from GeneChem Company (Shanghai, China). Cells were infected with lentiviral supernatants for 24 h. Stable clonal cell lines were selected with 2 μg/mL puromycin (Sigma, USA). PHF8-siRNA was purchased from GenePharma (Suzhou, China). Cells were transfected with PHF8-siRNAs for 48 h. Details are provided in Supplementary Table 3.

### Western blotting

Total cellular protein was isolated from cells using RIPA buffer (25 mM Tris-HCl, pH 7.6, 150 mM NaCl, 1 mM EDTA and 1% NP-40) supplemented with cocktail. Protein concentration was determined by G-250 assay. Equal amounts of protein were separated by SDS-polyacrylamide gel electrophoresis. β-actin served as a loading control. Detailed information for all antibodies used is provided in Supplementary Table 5.

Full and uncropped western blots are included in the Supplementary part.

### Co-immunoprecipitation

Cells were washed three times in cold PBS before being lysed in IP lysis buffer (20 mM Tris-HCl, pH 7.5, 150 mM NaCl, 1% Triton X-100, supplemented with protease inhibitor, 2 mL) supplemented with protease and phosphatase inhibitors (1:1000). Total protein lysate (2 mg) was used for each IP using specific antibodies, Protein A/G agarose beads (GE Healthcare Uppsala, Sweden) were added to the cells and incubated overnight at 4 °C. Washed precipitated proteins were analyzed by Western blotting. Detailed information for all antibodies used is provided in Supplementary Table 5.

### GST pull-down

Details can be found in the Supplemental Materials and Methods of Supplementary Information. The GST pull-down assay used in this study has been described previously in detail [59, 60].

### IP and mass spectrometry

Details can be found in the Supplemental Materials and Methods of Supplementary Information. The method of the mass spectrometry experiments has been described previously in detail [61].

### RNA-seq and RIP-seq analysis

mRNA was collected from each group of cells using Trizol Reagent (1 mL, Invitrogen, USA), and three replicate experiments were set up for each group. Following UID-mRNA-seq detection, differential alternative splicing (AS) analysis was performed using rMATS software. Data were supported by Wuhan Kangjie Technology Co., Ltd. (Wuhan, China). The SW480 cell pellet was collected, and the corresponding RNA-protein complex was precipitated with RBM39 antibody, followed by isolation and purification, and the RNA bound to the complex was sequenced and analyzed. Data were supported by Wuhan Kangjie Technology Co., Ltd.

### RNA isolation and RT-qPCR

Total RNAs were extracted using Trizol Reagent (500 μL, Invitrogen, USA) and converted into cDNAs using the PrimeScript RT Reagent Kit (TaKaRa, Japan). Quantitative PCR (qPCR) was undertaken using SYBR Premix Ex TaqII (Takara, Japan). The primers used for qPCR are shown in Supplementary Table 4. All results were normalized to β-actin and presented as fold induction relative to the control.

### RNA immunoprecipitation (RIP)

Details can be found in the Supplemental Materials and Methods of Supplementary Information.

### Transwell migration and invasion assay

For in vitro migration assays, SW480, SW620, or HT-29 cells (5 × 10^4^ cells in 100 mL RPMI-1640 medium without FBS) were seeded on top of a transwell chamber (Bedford, MA, USA). The lower chamber was supplemented with 600 mL RPMI-1640 medium containing 10% fetal bovine serum. After 24 h, cells that had not migrated from the upper membrane were wiped off with a cotton swab, and those that migrated from the lower membrane surface were fixed, stained, photographed, and counted. In the cell invasion assay in vitro, the upper chamber of transwells was treated with 10% matrix gel, and the other steps were the same as above.

### Dual-luciferase reporter assay

SW480 cells were seeded in 12-well plates and luciferase reporter plasmids were co-transfected with plasmids such as CDK5RAP2 S and PHF8 using Lipofectamine 3000 (Invitrogen, Carlsbad, CA, USA). Luciferase activity was detected 48 h after transfection using a Dual-Luciferase Reporter Assay System (Promega, USA).

### Chromatin immunoprecipitation (ChIP)

Details can be found in the Supplemental Materials and Methods of Supplementary Information.

### Animal models

Six-week-old female BALB/c nude mice (Spavo Biotechnology Co. Ltd., Beijing, China) were injected into the tail vein with 2 × 10^6^ SW480-luc cells with stable expression of shCtrl, shCDK5RAP2, shCDK5RAP2+L and shCDK5RAP2+S (five mice per group). The animals were imaged weekly for 8 weeks using a Carestream MS FX Pro in vivo imaging system (Carestream Health, Rochester, NY, USA). For in vivo fluorescence imaging, mice first received an intraperitoneal injection of the substrate and then were anesthetized with isoflurane 10 min later; whole-body images were then acquired. The data were analyzed using Carestream MI image analysis software, and fluorescence signals were normalized. After the final imaging session, the mice were sacrificed, and the organs (lung, liver, kidney, stomach, spleen and intestine) were examined for metastasis.

### Clinical samples

Samples include pairs of fresh ice-frozen colorectal and adjacent non-cancerous tissue samples (*n* = 75); serial sections of adjacent colorectal tissue samples (*n* = 30), primary colorectal cancer without metastasis tissue samples (*n* = 60) and primary colorectal cancer with lymph node and distant metastasis tissue samples (*n* = 40). Samples were obtained from the Department of Gastrointestinal Surgery of the First Affiliated Hospital of China Medical University. Detailed information for tissue samples is provided in Supplementary Table 6.

### Immunohistochemistry (IHC)

Details can be found in the Supplemental Materials and Methods of Supplementary Information.

### Statistical analysis

We used GraphPad Prism 8 software for statistical analysis. Correlations of immunohistochemical scores were analyzed using Pearson’s test, and other data were analyzed using Student’s *t*-test. The *χ*^2^ test was used to analyze the association between MORC2 or RBM39 expression and clinical features. Data are presented as mean ± SD of at least three independent experiments. Statistical significance was assigned when *P* values were <0.05. Specifically, *P < 0.05, **P < 0.01, ***P < 0.001, ****P < 0.0001, not significant (n.s) when *P* > 0.05.

### Supplementary information

Supplementary Information

Original Data File

## Data Availability

Materials are available upon request. MORC2 and CDK5RAP2 mass spectrometry data have been deposited at Figshare.com, the DOI number were 10.6084/m9.figshare.25522639 (https://figshare.com/articles/dataset/MORC2_proteomic/25522639) and 10.6084/m9.figshare.25522810 (https://figshare.com/articles/dataset/CDK5RAP2_proteomic/25522810). RBM39 RIP-seq, shMORC2 RNA-seq and shRBM39 RNA-seq data that support the findings of this study have been deposited in GEO with the accession code GSE246656, GSE246909 and GSE246915.
