# Supplementary material for: MORC2 regulates RBM39-mediated CDK5RAP2 alternative splicing to promote EMT and metastasis in colon cancer
Source: Cell Death Dis. 2024 Jul 24;15(7):530. doi: 10.1038/s41419-024-06908-y (PMC11269669; doi:10.1038/s41419-024-06908-y)
Supplement: Supplementary file 1 — Supplementary Information [file 41419_2024_6908_MOESM1_ESM.pdf]

Supplemental Materials and Methods

GST pull-down

After expanding pGEX-4T-2-RBM39, it was induced with 1 mM IPTG at 30 °C for 3 h. Lysates (20% glucose, 10% glycerol, 2 mM $MgCl_2$, 50 mM Tris, pH 8.0) were added to the pellet after centrifugation. GST protein was purified by incubating the supernatant with beads after sonication disruption. At the same time, eukaryotic plasmid (Flag-MORC2) was subjected to TNT reaction (Promega, America). Purified Flag-MORC2 protein and GST fused protein were incubated in pull-down buffer (20 mM Tris, pH 7.5, 50 mM NaCl, 10% glycerol, 1% NP-40). After incubation, the beads were pelleted and washed with the pull-down buffer, followed by elution of proteins. Protein samples were analyzed by Western blotting. The GST pull-down assay used in this study has been described previously in detail [59-60].

IP and Mass spectrometry

Flag-vector and Flag-MORC2 were stably overexpressed in HCT116 cells. These cells were harvested and lysed with lysis buffer (20 mM Tris-HCl (pH 7.5), 150 mM NaCl, 1% Triton X-100, supplemented with protease inhibitor). Then, the cellular extracts were subjected to affinity purification using anti-Flag affinity columns. The Flag peptide-eluted material was resolved on an 8% SDS-PAGE gel. The gels were stained with Coomassie brilliant blue (Invitrogen) after electrophoresis. The bands were excised from the gel and subjected to mass spectrometry (MS) analysis. The mass spectrometry experiments and data analysis were supported by Guangzhou Kidio Biotechnology Co., Ltd (Guangzhou, China).

After washing with ultrapure water, the SDS-PAGE gel was cut into small pieces of 1 mm square and put in an Eppendorf tube. 500 μL of destaining solution which was consisted of 50 mM ammonium bicarbonate (NH_4HCO_3) and 50% acetonitrile (ACN) was used for decolorizing until the gel pieces became colorless at 37 °C, 600 rpm. After washing with 50 mM NH_4HCO_3, the gel pieces were solidified with ACN. Then the gel pieces were reduced with 10 mM dithiothreitol (DTT) which was dissolved with 50 mM NH_4HCO_3 at 37 °C, 500 rpm for 1 h. After cooling to room temperature, ACN was added to solidify the gel. 55 mM iodoacetamide (IAA) dissolved with 50 mM NH_4HCO_3 was added to gel to alkylate sample at room temperature for 40 min in darkness. After washing with 50 mM NH_4HCO_3, the gel pieces were solidified with ACN. Appropriate volume of 13 ng/μL trypsin solution dissolved with 50 mM NH_4HCO_3 was added to digest gel pieces and incubated at 37 °C overnight. Trifluoroacetic acid (TFA) was added to the sample to the final concentration of 1% to terminate the digestion process. At last, 0.1% TFA with 60% ACN and 0.1% TFA with 90% ACN were used to extract peptides from gel pieces. Peptides were desalted by Pierce C18 Spin Tips, and dried in a speed vacuum concentrator.

The peptides were re-dissolved in solvent A (A: 0.1% formic acid in water) and analyzed by Q-Exactive Plus coupled to an EASY-nanoLC 1200 system (Thermo Fisher Scientific, MA, USA). 4 μL peptide sample was loaded onto a 25 cm analytical column (75 μm inner diameter, 1.9 μm resin (Dr. Maisch) and separated with 60 min gradient starting at 2%

buffer B (80% ACN with 0.1% FA) followed by a stepwise increase to 30% in 47 min, 100% in 1 min and stayed there for 12 min. The column flow rate was maintained at 300 nL/min with a column temperature of 40 °C. The electrospray voltage was set to 2 kV. The mass spectrometer was run under data dependent acquisition (DDA) mode, and automatically switched between MS and MS/MS mode. The survey of full scan MS spectra (m/z 350-1,800) was acquired in the Orbitrap with 70,000 resolution. The automatic gain control (AGC) target of 3e6 and the maximum injection time of 50 ms. Then the precursor ions were selected into collision cell for fragmentation by higher-energy collision dissociation (HCD), the normalized collection energy was 28. The MS/MS resolution was set at 17,500, the automatic gain control target of 1e5, the maximum injection time of 45 ms, and dynamic exclusion was 30 seconds.

Tandem mass spectra were processed by PEAKS Studio version 10.6 (Bioinformatics Solutions Inc., Waterloo, Canada). The database was Ensembl release 90 GRCh38. The protein quantification software was MaxQuant_1.5.3.30. Trypsin was set as the digestion enzyme, and Semi-specific was specified as the digest type. PEAKS DB was searched with a fragment ion mass tolerance of 0.02 Da and a parent ion tolerance of 10 ppm. The max missed cleavages was 2. Carbamidomethyl on Cysteine was specified as the fixed modification. Oxidation on Methionine, Deamination on Asparagine and Glutamine, and Acetylation on protein N-term were specified as the variable modifications. The peptides with 1% FDR and the proteins with 1% FDR and containing at least 1 unique peptide were filtered.

Flag-vector, Flag-CDK5RAP2 L or Flag-CDK5RAP2 S were transfected into SW620 cells, and the Co-IP experimental process was the same as above. The bands were excised from the gel and subjected to mass spectrometry analysis. The mass spectrometry experiments and data analysis were supported by Shanghai Applied Protein Technology Co., Ltd (Shanghai, China). The method of the mass spectrometry experiments has been described previously in detail [61].

RNA immunoprecipitation (RIP)

The Magna RIP RBP immunoprecipitation kit (EMD Millipore, USA) was used for RIP procedures. Cells were first lysed with RIP lysis buffer, and supernatants were incubated with IgG, MORC2, RBM39, or Flag antibodies (5 μg) with immunoprecipitated protein A/G beads. Complexes were then eluted with 900 μL RIP immunoprecipitation buffer (860 μL RIP wash buffer, 35 μL 0.5 M EDTA, 5 μL RNase inhibitor) and purified with 150 μL proteinase K buffer (117 μL RIP wash buffer, 15 μL 10% SDS, 18 μL proteinase K). Finally, CDK5RAP2 in the extracted RNA was detected by RT-qPCR. The primers used for qPCR are shown in Supplementary Table 4.

Chromatin immunoprecipitation (ChIP)

Cells were cross-linked with 1% formaldehyde for 10 min and neutralized with 0.125 M glycine. Cells were disrupted by sonication, and protein-DNA complexes were precipitated with Flag, Myc, IgG, or PHF8 antibodies (5 μg). The complexes were washed in the order of low-salt wash buffer (20 mM Tris, pH 8.1, 150 mM NaCl, 2 mM EDTA, 0.1% SDS, 1% Triton X-100, 1 time), high-salt wash buffer (20 mM Tris, pH 8.1, 500 mM NaCl, 2 mM EDTA, 0.1% SDS, 1% Triton X-100, 1 time), LiCl wash buffer (10 mM Tris, pH 8.1, 1 mM EDTA, 250 mM LiCl, 1% NP-40, 1% Deoxycholic Acid,

1 time), TE wash buffer (10 mM Tris, pH 8.1, 1 mM EDTA, 2 times) and then eluted with SDS buffer (20% SDS, 100 mM $NaHCO_3$). qPCR was carried out with primers specific to the slug promoter region. The primers are shown in Supplementary Table 4.

Immunohistochemistry (IHC)

The UltraSensitive IHC Assay kit (Maixin Biotechnology Co., Ltd., China) was used for IHC procedures. Dewaxing and rehydration were performed in the order of xylene, 100% ethanol, 95% ethanol, 90% ethanol, 80% ethanol, 70% ethanol, followed by antigen retrieval with citrate solution (0.01 M citrate solution, pH 6.0) and incubation with MORC2, RBM39, E-cadherin or Slug antibodies (1:200) overnight at 4 °C. Detailed information for all antibodies used is provided in Table S3. Secondary antibody incubated at room temperature and visualized with DAB. Then the nucleus was stained with hematoxylin (Maixin Biotechnology Co., Ltd., China). After dehydration (70%, 80%, 90%, 95%, 100% ethanol) and transparentization (xylene), the sections were sealed with neutral resins and photographed. The staining intensity was graded as 0 (no color), 1 (light yellow), 2 (light brown), or 3 (brown), and the number of positive cells was graded as 0 (< 5%), 1 (5%–25%), 2 (25%–50%), 3 (51%–75%), or 4 (> 75%). The two grades were multiplied. The score was 0-12. We divided them into two groups: 0-4 was the low expression group and 6-12 was the high expression group.

Supplemental Figures

Supplementary Fig. S1

A

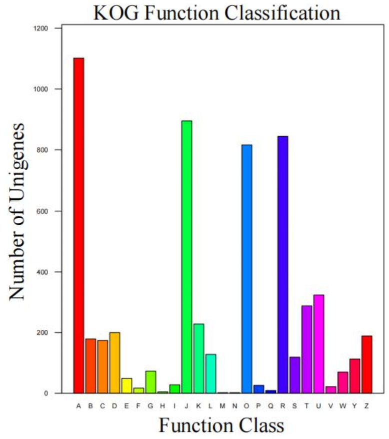

A: RNA processing and modification
B: Chromatin structure and dynamics
C: Energy production and conversion
D: Cell cycle control, cell division, chromosome partitioning
E: Amino acid transport and metabolism
F: Nucleotide transport and metabolism
G: Carbohydrate transport and metabolism
H: Coenzyme transport and metabolism
I: Lipid transport and metabolism
J: Translation, ribosomal structure and biogenesis
K: Transcription
L: Replication, recombination and repair
M: Cell wall/membrane/envelope biogenesis
N: Cell motility
O: Posttranslational modification, protein turnover, chaperones
P: Inorganic ion transport and metabolism
Q: Secondary metabolites biosynthesis, transport and catabolism
R: General function prediction only
S: Function unknown
T: Signal transduction mechanisms
U: Intracellular trafficking, secretion, and vesicular transport
V: Defense mechanisms
W: Extracellular structures
Y: Nuclear structure
Z: Cytoskeleton

B

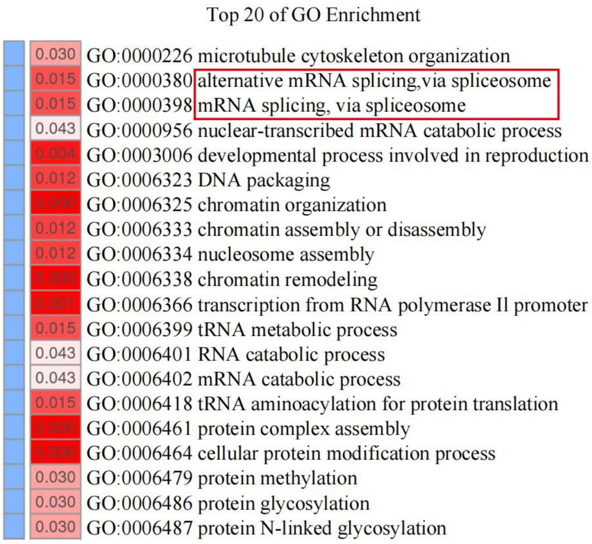

C

Symbol	Intensity MORC2
HNRNPM	956840000
PTBP1	632330000
SRSF3	541510000
HNRNPF	373430000
SRSF1	337650000
SRSF2	327430000
YBX1	237740000
SRSF5	232390000
DHX15	112080000
HNRNPC	105300000
RBM39	104750000
U2AF2	101680000
DROSHA	74636000
IQGAP1	74186000
SNRPD3	72582000
CAD	72446000
IGF2BP2	54659000
CDC5L	51516000
SLC8A1	45144000
EIF4A3	41304000

D

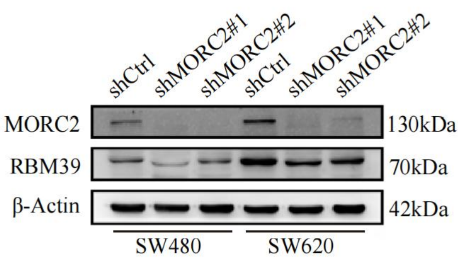

E

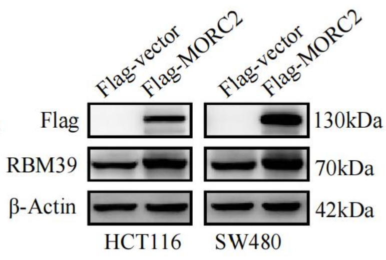

F

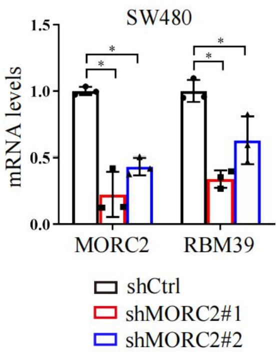

G

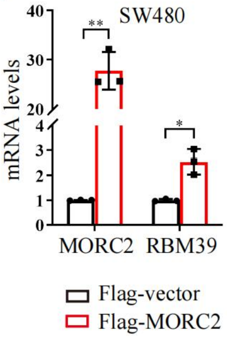

Fig. S1 Analysis of MORC2-binding proteins and the expression of RBM39 regulated by MORC2.

A,B KOG (A) and GO (B) analysis of MORC2-binding proteins.

C Top 20 splicing factors interacting with MORC2 by MS analysis were ranked by intensity.

D Western blot analysis of the indicated protein expression in SW480 and SW620 cells after knockdown of MORC2.

E Western blot analysis of the indicated protein expression in HCT116 and SW480 cells transfected with Flag-vector and Flag-MORC2.

F RT-qPCR was used to detect the effect of MORC2 knockdown on RBM39 mRNA expression. n=3, **P*<0.05.

G SW480 cells were transfected with Flag-vector and Flag-MORC2, followed by RT-qPCR to detect the mRNA level of RBM39. n=3, **P*<0.05, ***P*<0.01.

Supplementary Fig. S2

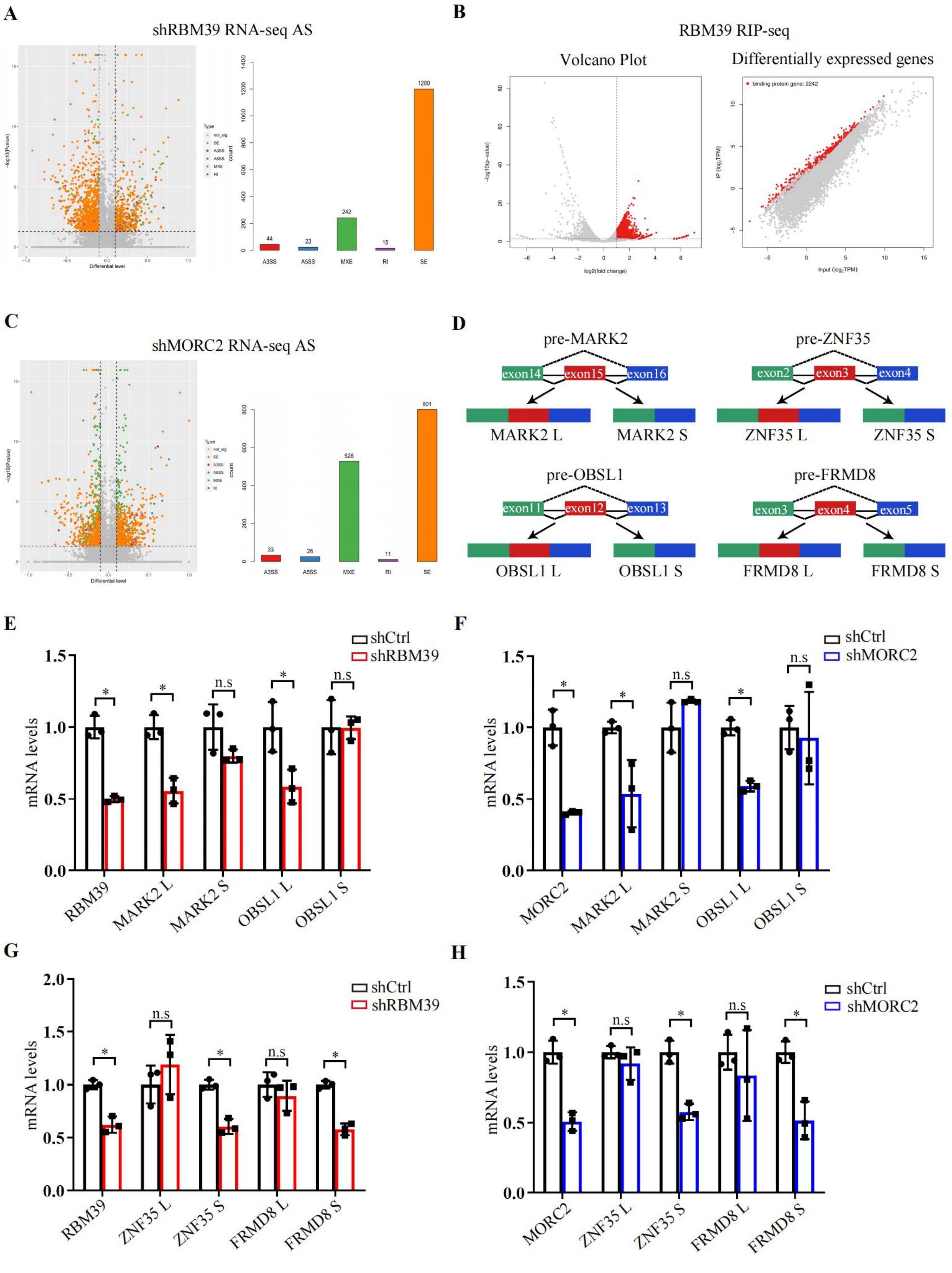

Fig. S2 Alternative splicing genes regulated by both RBM39 and MORC2.

A and C RNA-seq data analysis showed alternative splicing events regulated by RBM39 silencing (A) and MORC2 silencing (C), respectively. Volcano plot on the left and statistical plot of the number of splicing events on the right, with three independent replicate experiments.

B Volcano plot showing RNA bound to RBM39 in RIP-seq data (red).

D Schematic representation of pre-MARK2, pre-OBSL1, pre-ZNF35 and pre-FRMD8 alternative splicing. Exons were represented by green, red and blue rectangles, introns by black lines, and splicing patterns by dashed lines.

E,F RT-qPCR was used to detect the effect of RBM39 knockdown (E) or MORC2 knockdown (F) on MARK2 L, MARK2 S, OBSL1 L and OBSL1 S mRNA expression. n=3, *$P<0.05$, n.s indicates no significance.

G,H RT-qPCR was used to detect the effect of RBM39 knockdown (G) or MORC2 knockdown (H) on ZNF35 L, ZNF35 S, FRMD8 L and FRMD8 S mRNA expression. n=3, *$P<0.05$, n.s indicates no significance.

Supplementary Fig. S3

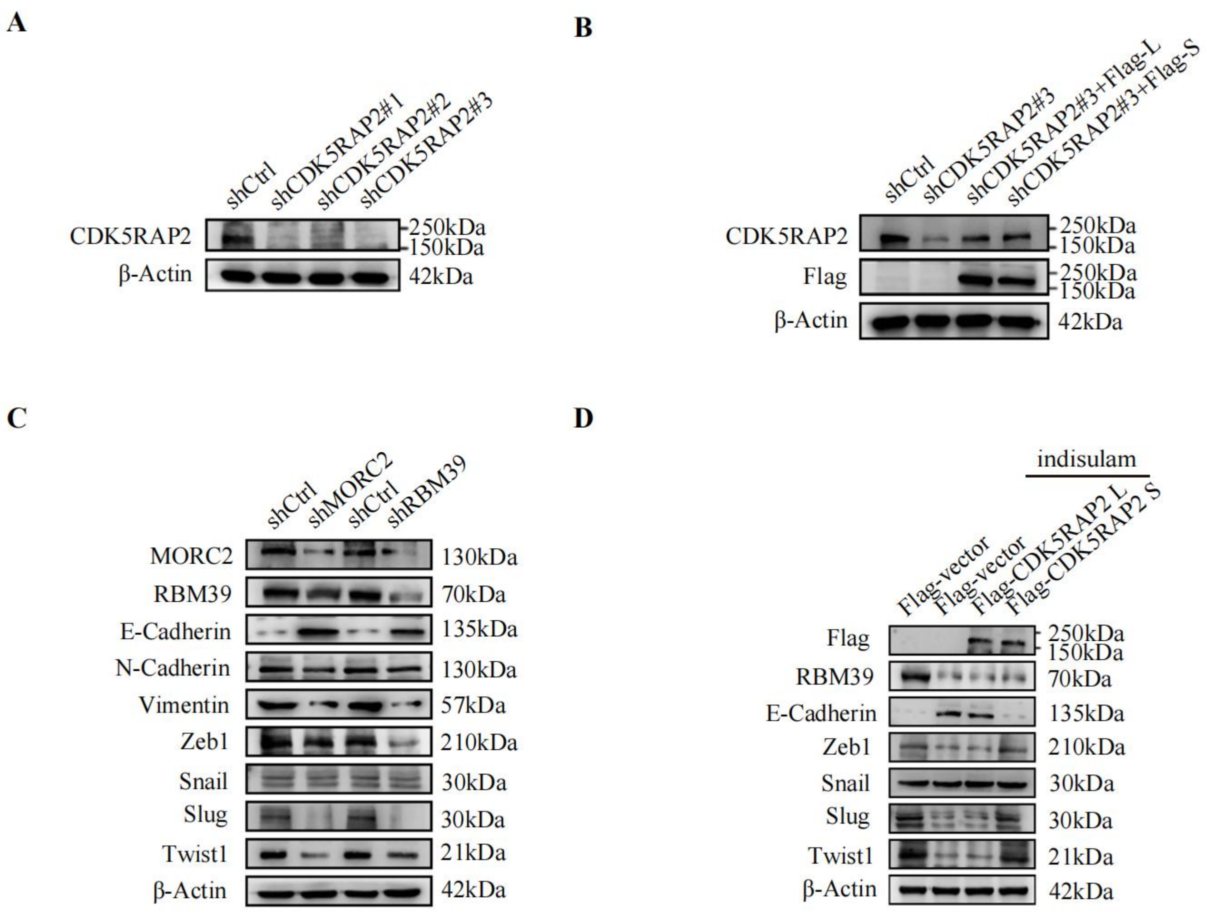

Fig. S3 MORC2 and RBM39 promote EMT of colon cancer cells.

A,B Stable CDK5RAP2 silencing SW480-luc cell lines were constructed by lentivirus-mediated shRNA, and western blot was used to detect CDK5RAP2 expression (A). Based on CDK5RAP2 knockdown cells (shCDK5RAP2#3), stable CDK5RAP2 L (shCDK5RAP2#3 + Flag-L) or CDK5RAP2 S (shCDK5RAP2#3 + Flag-S) overexpression cell lines were constructed by lentiviral infection, western blot was used to detect CDK5RAP2 expression (B).

C Western blot analysis of the indicated protein expression in SW620 cells after knockdown of MORC2 or RBM39.

D Western blot analysis of the indicated protein expression in SW480 cells transfected with Flag-vector, Flag-CDK5RAP2 L and Flag-CDK5RAP2 S treated by indisulam or not.

Supplementary Fig. S4

Symbol	Abundances	Symbol	Abundances
SRSF6	516674891.5	TF	9229543
UTY	147102448	COL4A4	9129127
RBM42	119530464.9	RNH1	6892056
ROS1	18405084	PHF8	6211977.5
ZRSR2	18211976	SRPK1	5699964.5
PDS5B	16260709	AP2B1	5365967.2
KIAA1549L	15323272	CLPB	4900988
RAB11A	12743687	POP1	4799666.5
DNAH10	12497474	TRIM41	4701743.5
TLK2	11133803.8	IQCE	4575859
NANS	10746723	DDOST	4524424.6
PCBP3	10654685	WASHC4	4466407
ASCC2	10395770.4	CEP85L	4081164.8
BT3.3	9948699	EIF4A2	4026373.8
NACA	9449798	PB1	4026143.5

Fig. S4 Top 30 proteins interacting with CDK5RAP2 S.

Top 30 proteins interacting with CDK5RAP2 S by MS analysis were ranked by abundances.

Supplemental Tables

Supplementary Table 1： Information of 26 skipped exon events regulated upon RBM39 and MORC2 in shRBM39 RNA-seq.

Gene name	AS type	AS feature	Skipped exon	Skipped exon length	GC content	Previous/ downstream intron length	*P* value
MTSS1L	SE	70679314-70679323	7	9 bp	44.44%	306 bp/905 bp	1.61E-12
CHAF1A	SE	4408902-4409759	3	857 bp	53.91%	2,940 bp/8,260 bp	1.04E-11
PLEC	SE	143922503-143925884	31	3,381 bp	70.98%	899 bp/108 bp	9.40E-09
MARK2	SE	63903985-63904147	15	162 bp	69.14%	827 bp/638 bp	0.000008
DAXX	SE	33321718-33321977	2	256 bp	57.42%	887 bp/151 bp	0.000033
PPP1R18	SE	30684407-30686046	1b	1,639 bp	62.23%	-/5,018 bp	0.000292
PI4KB	SE	151326171-151326373	2	202 bp	48.51%	897 bp/9,662 bp	0.000318
ASXL1	SE	32429900-32430053	8	153 bp	71.24%	469 bp/1,267 bp	0.00032
FAM95C	SE	38541455-38541562	-	-	-	-	0.000416
ZNF35	SE	44652556-44652701	3	145 bp	51.72%	1,297 bp/5,999 bp	0.001031
SCRIB	SE	143807551-143807614	16	63 bp	53.97%	994 bp/538 bp	0.002241
CDK5RAP2	SE	120407011-120407248	32	237 bp	60.34%	1,098 bp/2,898 bp	0.002824
RGS19	SE	64076856-64076954	2	98 bp	67.35%	2,918 bp/210 bp	0.002863
MTA1	SE	105449358-105449409	4	51 bp	66.67%	3,847 bp/648 bp	0.003497
GORASP1	SE	39102677-39102881	3	204 bp	59.31%	591 bp/1,575 bp	0.003668
TCF20	SE	42168608-42168736	5	128 bp	64.84%	1,110 bp/7,250 bp	0.004238
CTD-2270P14.1	SE	2671594-2672570	-	-	-	-	0.007867
GOLGA2P7	SE	84204176-84204331	4	155 bp	62.09%	94 bp/90 bp	0.008723
AXIN1	SE	291189-291297	9	108 bp	67.59%	2,190 bp/1,582 bp	0.011628
FRMD8	SE	65393572-65393674	4	102 bp	65.69%	4,044 bp/366 bp	0.012389
FRMD4A	SE	13663452-13663509	19	57 bp	49.12%	2,587 bp/2,899 bp	0.018825
ADGRB2	SE	31742837-31743002	7	165 bp	72.73%	1,190 bp/620 bp	0.022458
SBF1	SE	50457033-50457111	29	78 bp	71.79%	2,143 bp/360 bp	0.030108
BRF1	SE	105241264-105241414	6	150 bp	62.67%	11,092 bp/12,351 bp	0.036708
OBSL1	SE	219557342-219557618	12	276 bp	71.38%	204 bp/619 bp	0.044494
ARVCF	SE	19971215-19971335	19	120 bp	70.00%	550 bp/472 bp	0.044776

Supplementary Table 2： Information of 26 skipped exon events regulated upon RBM39 and MORC2 in shMORC2 RNA-seq.

Gene name	AS type	AS feature	Skipped exon	Skipped exon length	GC content	Previous/ downstream intron length	*P* value
SBF1	SE	50457033-50457111	29	78 bp	71.79%	2,143 bp/360 bp	9.93E-07
SCRIB	SE	143807551-143807614	16	63 bp	53.97%	994 bp/538 bp	0.000001
MTSS1L	SE	70679314-70679323	7	9 bp	44.44%	306 bp/905 bp	0.000003
GORASP1	SE	39102677-39102881	3	204 bp	59.31%	591 bp/1,575 bp	0.000029
PI4KB	SE	151326171-151326373	2	202 bp	48.51%	897 bp/9,662 bp	0.000049
ADGRB2	SE	31728024-31728081	32	57 bp	70.18%	100 bp/419 bp	0.000762
GOLGA2P7	SE	84204599-84204691	-	-	-	-	0.001409
CTD-2270P14.1	SE	2671594-2672570	-	-	-	-	0.002098
AXIN1	SE	346147-347106	2	959 bp	55.16%	5,262 bp/31,464 bp	0.002902
PLEC	SE	143922503-143925884	31	3,381 bp	70.98%	899 bp/108 bp	0.003555
FAM95C	SE	38541455-38541562	-	-	-	-	0.005982
ASXL1	SE	32429900-32430053	8	153 bp	71.24%	469 bp/1,267 bp	0.006347
CDK5RAP2	SE	120407011-120407248	32	237 bp	60.34%	1,098 bp/2,898 bp	0.006897
FRMD4A	SE	13663452-13663509	19	57 bp	49.12%	2,587 bp/2,899 bp	0.007784
DAXX	SE	33321718-33321977	2	256 bp	57.42%	887 bp/151 bp	0.008007
ARVCF	SE	19990584-19990812	3	228 bp	67.54%	19,642 bp/8,493 bp	0.012422
MARK2	SE	63903985-63904147	15	162 bp	69.14%	827 bp/638 bp	0.013249
MTA1	SE	105449358-105449409	4	51 bp	66.67%	3,847 bp/648 bp	0.014752
RGS19	SE	64076856-64076954	2	98 bp	67.35%	2,918 bp/210 bp	0.01981
CHAF1A	SE	4408902-4409759	3	857 bp	53.91%	2,940 bp/8,260 bp	0.026618
OBSL1	SE	219557342-219557618	12	276 bp	71.38%	204 bp/619 bp	0.032607
ZNF35	SE	44652556-44652701	3	145 bp	51.72%	1,297 bp/5,999 bp	0.0336
BRF1	SE	105241264-105241414	6	150 bp	62.67%	11,092 bp/12,351 bp	0.035482
TCF20	SE	42168608-42168736	5	128 bp	64.84%	1,110 bp/7,250 bp	0.035637
FRMD8	SE	65393572-65393674	4	102 bp	65.69%	4,044 bp/366 bp	0.041894
PPP1R18	SE	30684407-30686046	1b	1,639 bp	62.23%	-/5,018 bp	0.044372

Supplementary Table 3：Information of plasmids, lentivirus and siRNA.

Plasmids	Source	Identifier
Flag-MORC2	This paper	N/A
His-RBM39	Sino Biological	Cat# HG19470-NH
His-RBM39-△RS	Sangon Biotech	N/A
His-RBM39-△RRM1	Sangon Biotech	N/A
His-RBM39- △RRM2	Sangon Biotech	N/A
His-RBM39-△UHM	Sangon Biotech	N/A
pGEX-4T-2-RBM39	This paper	N/A
Flag-RBM39-FL	This paper	N/A
Flag-RBM39-△RS	This paper	N/A
Flag-RBM39-△RRM1	This paper	N/A
Flag-RBM39- △RRM2	This paper	N/A
Flag-RBM39-△UHM	This paper	N/A
Flag-CDK5RAP2 L	Sino Biological	Cat# HG30038-NF
Flag-CDK5RAP2 S	Sino Biological	Cat# HG19407-NF
CDK5RAP2 minigene	BGI	N/A
Myc-PHF8	Sino Biological	Cat# HG19177-NM
Slug promoter reporter	gift from Dr. Ceshi Chen	N/A
**Lentivirus and siRNA**	**Source**	**Identifier**
Lentivirus	GeneChem	N/A
siPHF8	GenePharma	N/A

Supplementary Table 4：Primers used for quantification of mRNA and genome DNA.

RT-primers	Primer sequence
RBM39-F	GTTCGTCGATGTTAGCTCAGTGC
RBM39-R	AGCCTCATAGGTCCAGCACTTC
MORC2-F	GGAGGTTCCTTCTCCCAAAGTC
MORC2-R	CAGAAACTGCGACACTCCGCTT
CDK5RAP2-F	ACCGATCAACACTGCACTCAGC
CDK5RAP2-R	GGATTGGCAAGCGGGACTTCTT
CDK5RAP2 L(S)-F1	TGGAACGGCAAGGATCTGAA
CDK5RAP2 L(S)-R1	TCACTGCCTGGGAGGAATCA
CDK5RAP2 L(S)-F2	GGTGCAGGAGGAGGTGAAGTTGAGG
CDK5RAP2 L(S)-R2	CTGATTTTGGTGTCACTGCCTGGGA
CDK5RAP2 L-F	CTCTGCAGAGCAGGCTCAAGG
CDK5RAP2 L-R	CTGATTTTGGTGTCACTGCCT
CDK5RAP2 S-F	GACTGAGAGAGGCGTCGGGAG
CDK5RAP2 S-R	GGAGACTGAGAGAGGCGTCGG
MARK2 L-F	CCTAACCATGCCAGGGTCCCG
MARK2 L-R	GGGAGGCAACAGGGACACGCT
MARK2 S-F	CAAAGACAGCACAGCCCCCCA
MARK2 S-R	TCACACCGTAGGGCAAATTCT
OBSL1 L-F	AGACGAGGGTTCGGAGCACCC
OBSL1 L-R	CGCACAGGTACTCCCCAGCGT
OBSL1 S-F	AGGTGGCTGAGCCACTGCTGG
OBSL1 S-R	TTGAACCATTCTGGGCCATCT
ZNF35 L-F	GGTCAGAACATATCCTGGGAT
ZNF35 L-R	CTAGAAAGTTCATGGTCCCAT
ZNF35 S-F	GAAGAAAAGGTGCTGAAACCA
ZNF35 S-R	TTCTCTCTTTATTCTCTGCCT
FRMD8 L-F	AGGTGCAGCTGAAACCCAAGC
FRMD8 L-R	CTGGAGCTCCCGCCGCTTTGG
FRMD8 S-F	CTCTGCTGGATGAGCCTTTCC
FRMD8 S-R	CTGGAGCTCCCGCCGCTTTGG

Slug-F	ATCTGCGGCAAGGCGTTTTCCA
Slug-R	GAGCCCTCAGATTTGACCTGTC
Snail-F	TGCCCTCAAGATGCACATCCGA
Snail-R	GGGACAGGAGAAGGGCTTCTC
Zeb1-F	GGCATACACCTACTCAACTACGG
Zeb1-R	TGGGCGGTGTAGAATCAGAGTC
Twist1-F	GCCAGGTACATCGACTTCCTCT
Twist1-R	TCCATCCTCCAGACCGAGAAGG
E-Cadherin-F	AGCCCCGCCTTATGATTCTC
E-Cadherin-R	TGCCCCATTCGTTCAAGTAGTC
N-Cadherin-F	AGCCAACCTTAACTGAGGAGT
N-Cadherin-R	GGCAAGTTGATTGGAGGGATG
PHF8-F	GGACACATACAGTCATCAGGCAC
PHF8-R	GGCTCTCATTTCCATCAAGGTCC
β-Actin-F	CACCATTGGCAATGAGCGGTTC
β-Actin-R	AGGTCTTTGCGGATGTCCACGT

**Construct primers**	**Primer sequence**
pGEX-4T-2-RBM39-F	CGCGGATCCATGGCAGACGATATTGAT
pGEX-4T-2-RBM39-R	CCGCTCGAGTCGTCTACTTGGAACCAG
Flag-RBM39-F	CGCGGATCCATGGCAGACGATATTGAT
Flag-RBM39-R	CCGCTCGAGTCGTCTACTTGGAACCAG
Flag-RBM39-△RS-F	AAAGAGGAAAAAAAGCAAGAGCTTTAGAGGCCGCTACAGAAGTC
Flag-RBM39-△RS-R	GACTTCTGTAGCGGCCTCTAAAGCTCTTGCTTTTTTTCCTCTTT
Flag-RBM39-△RRM1-F	AACTCCTGAGGAAAGAGATGCAGAAAAAAACAGAGCTGCAGCAA
Flag-RBM39-△RRM1-R	TTGCTGCAGCTCTGTTTTTTTCTGCATCTCTTTCCTCAGGAGTT
Flag-RBM39- △RRM2-F	ACAAAAGGGAAGTGCTGGACCTCGTACTGATGCTTCGAGTGCTA
Flag-RBM39- △RRM2-R	TAGCACTCGAAGCATCAGTACGAGGTCCAGCACTTCCCTTTTGT
Flag-RBM39-△UHM-F	AGAAGAAGTTGGATGGGATACCCCAACTTACCACAACCTGTTTC
Flag-RBM39-△UHM-R	GAAACAGGTTGTGGTAAGTTGGGGTATCCCATCCAACTTCTTCT

**CHIP-PCR-primers**	**Primer sequence**
Slug promotor P1-F	GATCTGTGCAGTGCACCCTC
Slug promotor P1-R	AGAAGAGTGTTAGACAATGTTTTGG

Slug promotor P2-F	TTGTTGTGAATAGACTGTGTAGAGT
Slug promotor P2-R	CAGTTTTCTAAGCTTGCTACTCTAT
Slug promotor P3-F	CCTCTGGCTTTTACTCCAGGTT
Slug promotor P3-R	GGGCACTCACAGATTTCCTAAG
Slug promotor P4-F	CACCTCACCCTCCAAACACA
Slug promotor P4-R	CTAAAAGAATAAGGAAAGAACAAAT
Slug promotor P5-F	CAAAAGATAGGGATAAAAGTCTGCA
Slug promotor P5-R	GCTAAAAAAGAAATTGAAGGATATT
Slug promotor P6-F	AGCATTATACAGGAAACTGGTAGAT
Slug promotor P6-R	TCTCTCACACTTTTGACAAGAGATC
Slug promotor P7-F	ATGTCCGGTGGTTCCAAAT
Slug promotor P7-R	TACTCAGGGCTTCCGCGAA
Slug promotor P8-F	TGAGTAGCGCAGCGCCCT
Slug promotor P8-R	ATGAGAGCCTATATTTGGAAGTGGC
Slug promotor P9-F	TAACACCAGAGGCTGGCCT
Slug promotor P9-R	TTTTCAAGAGAGGTAACCTCGCT
Slug promotor P10-F	TACTTAAACACTTTTTTTCCTCTCC
Slug promotor P10-R	AACTGAGCCCGTTTTGGCT

Supplementary Table 5:　Information of antibody and chemical reagent.

Antibodies	Source	Identifier
MORC2 (WB and Co-IP)	Abcam	Cat# ab14429; RRID: AB_301210
MORC2 (IHC)	NOVUS	Cat# NBP1-89295; RRID: AB_11036440
RBM39	Abcam	Cat# ab25801; RRID: AB_2936825
CDK5RAP2	Abcam	Cat# ab70213; RRID: AB_1209656
E-Cadherin	Proteintech	Cat# 20874-1-AP; RRID: AB_10697811
N-Cadherin	Proteintech	Cat# 22018-1-AP; RRID: AB_2813891
Vimentin	ZENBIO	Cat# R22775; RRID: AB_2895079
Zeb1	Proteintech	Cat# 21544-1-AP; RRID: AB_10734325
Snail	Proteintech	Cat# 13099-1-AP; RRID: AB_2191756
Slug (WB)	Proteintech	Cat# 12129-1-AP; RRID: AB_2191889
Slug (IHC)	ABclonal	Cat# A13352; RRID: AB_2760210
Twist1	Proteintech	Cat# 25465-1-AP; RRID: AB_2880093
PHF8	Abcam	Cat# ab280887; RRID: AB_2936826
H3K9me1	ABclonal	Cat# A2358; RRID: AB_2721265
H3K9me2	ABclonal	Cat# A2359; RRID: AB_2764319
H3K9me3	ABclonal	Cat# A2360; RRID: AB_2721266
H3K27me2	ABclonal	Cat# A2362; RRID: AB_2764322
H3K4me3	ABclonal	Cat# A2357; RRID: AB_2631278
H4K20me1	ACTIVE MOTIF	Cat# 39728; RRID: AB_2615074
Flag-tag (Co-IP)	Proteintech	Cat# 66008-4-Ig; RRID: AB_2918475
Flag-tag (WB)	Abmart	Cat# M20008; RRID: AB_2713960
His-tag (Co-IP)	Proteintech	Cat# 66005-1-Ig; RRID: AB_11232599
His-tag (WB)	GenScript	Cat# A00186; RRID: AB_914704
Myc-tag	GenScript	Cat# A00704; RRID: AB_914461
Lamin B1	Proteintech	Cat# 12987-1-AP; RRID: AB_2136290
β-Tubulin	Proteintech	Cat# 10094-1-AP; RRID: AB_2210695
β-Actin	Cell Signaling Technology	Cat# 3700; RRID: AB_2242334

Chemical reagent	Source	Identifier
DH5a Competent Cells	TaKaRa	Cat# 9057
BL21 Competent Cells	TaKaRa	Cat# 9126

Indisulam	MedChemExpress	Cat# HY-13650
Daminozide	MedChemExpress	Cat# HY-13643
D-Luciferin (potassium salt)	GLPBIO	Cat# GC43496
Magna RIP RBP immunoprecipitation Kit	Millipore	Cat# 17-700
TNT$^{@}$ Quick Coupled Transcription/Translation System	Promega	Cat# L1170
PrimeScript RT Reagent Kit	TaKaRa	Cat# RR037A
SYBR Premix Ex TaqII	TaKaRa	Cat# RR820A

Supplementary Table 6：Clinical colorectal cancer tissue sample information.

Number	Gender	Age	Tumor size（cm）	Differentiation	UICC	T	N	M	Metastasis
1	Male	63	2.5*2.5*2.5	Highly-Moderately	I	2	0	0	No
4	Female	88	5*5*5	Highly-Moderately	IIB	4b	0	0	No
5	Female	60	2.5*2.5*2.5	Moderately	II	3	0	0	No
8	Female	53	5*5*5	Moderately	IV	4b	2b	1	Liver
9	Male	87	4*4.5*4	Moderately	II	3	0	0	No
10	Male	62	5*5*5	Moderately	II	3	0	0	No
11	Female	80	4*4*4	Moderately	I	1	0	0	No
12	Female	84	6*5*6	Highly-Moderately	II	3	0	0	No
13	Male	52	4.5*4.5*4.5	Highly-Moderately	II	3	0	0	No
14	Male	79	3*3*3	Highly-Moderately	II	3	0	0	No
15	Male	68	4*4*4	Moderately-Poorly	III	3	2b	0	No
16	Male	46	7*7*7	Moderately	II	3	0	0	No
17	Male	72	2*2*2	Poorly	III	3	1a	0	No
19	Female	76	5*5*5	Moderately	I	2	0	0	No
20	Male	70	5*5*5	Moderately	III	3	1b	0	No
21	Female	63	4*4*4	Moderately	I	1	0	0	No
22	Female	32	4.5*4.5*4.5	Moderately	II	3	0	0	No
23	Female	81	6*6*6	Moderately	II	3	0	0	No
43	Male	71	2.5*2.5*2.5	Moderately-Poorly	III	2	1b	0	No
50	Female	64	5*5*5	Moderately	III	3	1c	0	No
51	Female	75	3.5*3.5*3.5	Moderately	III	3	1a	0	No
52	Female	62	3.5*3.5*3.5	Highly-Moderately	I	2	0	0	No
53	Male	56	5*5*5	Poorly	II	3	0	0	No
54	Female	72	1.5*1.5*1.5	Moderately	I	2	0	0	No
55	Female	65	2.5*2.5*2.5	Moderately	II	4b	0	0	No
56	Female	60	6*6*6	Moderately	II	3	0	0	No
57	Male	48	4.5*4.5*4.5	Moderately	I	2	0	0	No
58	Male	56	3.5*3*3.5	Highly-Moderately	I	2	0	0	No
59	Male	48	5*5*5	Moderately	III	4a	1a	0	No
60	Male	63	9*8*4	Moderately-Poorly	II	3	0	0	No
61	Female	42	9*9*9	Poorly	II	3	0	0	No

62	Female	64	3*3*3	Moderately	II	4a	0	0	No
63	Female	65	2.5*2.5*2.5	Moderately	II	3	0	0	No
64	Male	55	3*3*3	Moderately-Poorly	I	2	0	0	No
65	Male	65	7*7*7	Moderately-Poorly	I	2	0	0	No
66	Male	70	4*2.5*4	Highly-Moderately	II	3	0	0	No
67	Male	65	3*3*3	Moderately	II	3	0	0	No
68	Male	75	2*2*2	Moderately	I	2	0	0	No
69	Male	54	3*2*3	Moderately	II	3	0	0	No
70	Female	68	3*3*3	Highly	II	3	0	0	No
71	Female	50	6*6*6	Poorly	III	4a	2	0	No
72	Male	71	4*4*4	Moderately	II	3	0	0	No
73	Female	65	2.5*2.5*2.5	Moderately	I	2	0	0	No
74	Male	58	2*2*2	Moderately	II	3	1a	0	No
75	Female	65	2*2*2	Moderately	II	3	0	0	No
76	Female	73	3*3*3	Moderately	II	3	0	0	No
77	Male	54	3*3*3	Moderately-Poorly	III	3	2a	0	No
78	Male	65	6*4*6	Poorly	IV	3	1	1a	Liver
79	Male	60	4*4*4	Moderately-Poorly	IV	3	1	1a	Liver
80	Female	74	6*6*6	Moderately	III	3	2a	0	No
81	Female	81	5*4*4	Poorly	IV	4a	1b	1a	Liver
82	Male	55	6*6*6	Moderately	II	3	0	0	No
83	Male	66	5*3*5	Moderately	IV	2	0	1	Liver
84	Male	58	3*3*3	Moderately	IV	1	0	1a	Liver
85	Male	65	3*2.5*2	Moderately	III	3	1a	0	No
86	Female	71	6*5*6	Moderately	III	3	1	0	No
87	Male	69	2*2*2	Moderately	III	2	1	0	No
88	Male	54	2*2*2	Moderately	I	1	0	0	No
89	Female	52	4*4*4	Highly-Moderately	III	3	1	0	No
90	Female	56	3.5*3.5*3.5	Moderately	III	3	1	0	No
91	Male	71	4*4*4	Moderately	III	3	1	0	No
92	Female	59	4*4*4	Moderately	III	2	1	0	No
93	Female	79	12*12*12	Moderately-Poorly	III	3	2a	0	No
94	Female	79	7*5*7	Highly	II	3	0	0	No

95	Male	50	4.5*4.5*4.5	Moderately	I	2	0	0	No
96	Female	69	4*4*4	Moderately	I	2	0	0	No
97	Male	69	6*6*6	Highly	II	3	0	0	No
98	Female	63	3*5*3	Moderately	III	3	1	0	No
99	Male	52	7*7*7	Moderately-Poorly	II	3	0	0	No
100	Female	66	7*7*7	Moderately	III	3	1	0	No
101	Male	52	2*2*2	Moderately	II	3	0	0	No
102	Male	65	8*8*8	Highly-Moderately	I	2	0	0	No
103	Female	49	4*4*4	Highly-Moderately	II	3	0	0	No
104	Female	76	2*2*2	Moderately	IIIB	3	1a	0	No
105	Female	73	5*3.5*5	Moderately-Poorly	III	3	2b	0	No
106	Male	76	5*5*5	Moderately	I	2	0	0	No
107	Female	49	5*5*5	Highly-Moderately	IIC	4b	0	0	No
108	Male	69	5*5*5	Moderately-Poorly	III	3	1a	0	No
109	Female	66	4*4*4	Highly	IIIB	3	1a	0	No
110	Male	57	2*2*2	Moderately	I	1	0	0	No
111	Male	77	3*3*3	Moderately	III	3	1b	0	No
112	Female	77	6*6*6	Moderately	IIIC	3	2b	0	No
113	Female	47	3*3*3	Highly-Moderately	III	3	1b	0	No
114	Female	67	3*3*3	Poorly	I	2	0	0	No
115	Male	62	6*6*6	Moderately	III	1	1b	0	No
116	Male	77	7*4*7	Highly--Moderately	IIB	4a	0	0	No
117	Male	47	6*6*6	Moderately	III	1	1b	0	No
118	Male	67	11*11*11	Moderately-Poorly	II	3	0	0	No
119	Female	56	8*8*8	Moderately	II	3	0	0	No
120	Male	81	4*3.5*4	Moderately	III	3	1a	0	No
121	Male	84	4*4*4	Highly	II	3	0	0	No
122	Female	64	3*3*3	Moderately	I	2	0	0	No
123	Male	74	5*3*2.3	Moderately	III	3	1	0	No
124	Male	83	9*7*3.5	Moderately	IV	3	1b	1	Liver
125	Female	88	4*3*4	Moderately-Poorly	III	1	2b	0	No
126	Female	51	6*6*6	Moderately	II	3	0	0	No
127	Male	48	3*3*3	Moderately	I	2	0	0	No

128	Female	74	4*4*4	Moderately	III	3	1a	0	No
129	Female	76	4*4*4	Moderately	II	3	0	0	No
130	Male	64	4*4*4	Moderately	II	3	0	0	No
131	Male	87	3*3*3	Moderately	II	3	0	0	No
132	Male	62	3*3*3	Moderately-Poorly	II	3	0	0	No
133	Female	69	6*6*6	Moderately-Poorly	II	3	0	0	No
134	Female	59	4*4*4	Moderately	I	2	0	0	No
135	Female	67	2*2*2	Highly	I	2	0	0	No
136	Male	68	4*4*4	Moderately	II	3	0	0	No
137	Female	64	5*4*5	Moderately	I	2	0	0	No
138	Male	53	3.5*3.5*3.5	Moderately-Poorly	II	3	0	0	No
139	Female	50	0.3*0.3*0.3	Moderately-Poorly	II	3	0	0	No
140	Female	70	5*5*5	Moderately	II	3	0	0	No
141	Male	57	7*5*7	Moderately-Poorly	II	3	0	0	No
142	Female	61	3*3*3	Moderately-Poorly	IV	1	1	0	No
143	Female	65	5.5*5.5*5.5	Moderately--Poorly	I	1	0	0	No
144	Female	60	2.5*2.5*2.5	Moderately--Poorly	I	2	0	0	No
145	Female	60	2.5*2*2.5	Highly-Moderately	III	2	1b	0	No
146	Male	60	6*6*6	Moderately	III	3	1b	0	No
147	Male	62	5*5*5	Moderately	II	3	0	0	No
148	Female	69	3.5*3*3.5	Moderately	III	3	1b	0	No
149	Female	39	5*2*5	Moderately-Poorly	III	3	2	0	No
150	Male	69	3.5*2*3.5	Moderately-Poorly	III	3	1	0	No
151	Female	63	2.8*2.8*2.8	Highly-Moderately	II	3	0	0	No
152	Male	75	7*7*7	Moderately	III	2	2	0	No
153	Female	47	1.5*1.5*1.5	Poorly	I	2	0	0	No
154	Male	65	5.5*5.5*5.5	Moderately	I	2	0	0	No
155	Female	78	3*3*3	Moderately-Poorly	III	3	1b	0	No
156	Male	49	10.5*10.5*10.5	Moderately	I	2	0	0	No
157	Male	33	2.5*2.5*2.5	Moderately-Poorly	II	3	0	0	No
158	Female	71	5*5*5	Moderately	I	1	0	0	No
159	Female	65	2.5*1.5*2.5	Moderately	I	2	0	0	No
160	Male	64	2.5*1.5*2.5	Moderately-Poorly	I	2	0	0	No

161	Male	73	2*1.5*2	Moderately-Poorly	III	3	1a	0	No
162	Male	37	2*2*2	Moderately	II	3	0	0	No
163	Male	56	5*5*5	Moderately	II	3	0	0	No
164	Male	59	4*4*4	Moderately-Poorly	IV	4a	1a	1b	Peritoneum
165	Male	61	6*6*6	Highly-Moderately	I	2	0	0	No
166	Male	90	4*4*4	Highly-Moderately	II	3	0	0	No
167	Female	75	2.2*1.5*2.2	Moderately-Poorly	II	3	0	0	No
168	Female	47	5.5*4.5*5.5	Moderately-Poorly	II	3	0	0	No
169	Male	81	7.5*5.5*7.5	Moderately	I	1	0	0	No
170	Male	56	6.5*6.5*6.5	Highly-Moderately	IV	2	0	1	Liver
171	Male	71	1.5*4*1.5	Moderately	I	2	0	0	No
172	Male	57	4*3*4	Highly-Moderately	II	3	0	0	No
173	Female	80	4*2.5*4	Moderately	I	2	0	0	No
174	Male	60	4*4*4	Moderately	IIA	3	0	0	No
175	Female	45	4*2.5*4	Moderately-Poorly	III	3	1a	0	No
176	Male	52	4.5*4.5*4.5	Moderately	II	3	0	0	No
261	Female	64	8*8*8	Moderately	III	3	2a	0	No
262	Male	56	3*3*3	Moderately-Poorly	III	2	1a	0	No
263	Male	67	4.5*4*4.5	Moderately	III	3	1a	0	No

Supplementary Table 7: Abbreviations list.

Abbreviation	Full Name
ACN	acetonitrile
AML	myeloid leukemia
ArgBP2	Arg Kinase-binding protein 2
AS	alternative splicing
BIN1	bridging integrator 1
BLI	bioluminescence imaging
CAIX	carbonic anhydrase IX
CDK5RAP2	cyclin-dependent kinase 5 regulatory subunit associated protein 2
ChIP	chromatin immunoprecipitation
Co-IP	Co-Immunoprecipitation
CRC	colorectal cancer
DCAF15	DDB1 and CUL4 associated factor 15
DFS	disease free survival
DHX9	DExH-box helicase 9
DTT	dithiothreitol
ECL	enhanced chemiluminescence
EMT	epithelial-mesenchymal transition
FBS	fetal bovine serum
FRMD8	FERM domain containing 8
GST	glutathione-s-transferase
hnRNPM	Heterogeneous Nuclear Ribonucleoprotein M
IAA	iodoacetamide
IHC	immunohistochemistry
IPTG	isopropy1β-D-1-thiogalactopyranoside
MARK2	microtubule affinity regulating kinase 2
MORC2	MORC family CW-type zinc finger 2
NH_4HCO_3	ammonium bicarbonate
NONO	non-POU domain containing octamer binding
OBSL1	obscurin like cytoskeletal adaptor 1
PCM	pericentriolar matrix
PCNT	pericentrin
PUF60	poly(U) binding splicing factor 60

PHF8	PHD finger protein 8
RBM39	RNA binding motif protein 39
RBP	RNA-binding protein
RIP	RNA immunoprecipitation
SE	skipped exon
SF3B1	splicing factor 3b subunit 1
SFPQ	splicing factor proline and glutamine rich
Slug	snail family transcriptional repressor 2
SRSF6	serine and arginine rich splicing factor 6
Snail	snail family transcriptional repressor 1
TFA	Trifluoroacetic acid
TRIM27	tripartite motif containing 27
Twist1	twist family bHLH transcription factor 1
U2AF65	U2 small nuclear RNA auxiliary factor 2
Zeb1	zinc finger E-box binding homeobox 1
ZNF35	zinc finger protein 35
ZO-1	tight junction protein 1
