# Supplementary material for: MORC2 regulates RBM39-mediated CDK5RAP2 alternative splicing to promote EMT and metastasis in colon cancer
Source: Cell Death Dis. 2024 Jul 24;15(7):530. doi: 10.1038/s41419-024-06908-y (PMC11269669; doi:10.1038/s41419-024-06908-y)

Figure 1C

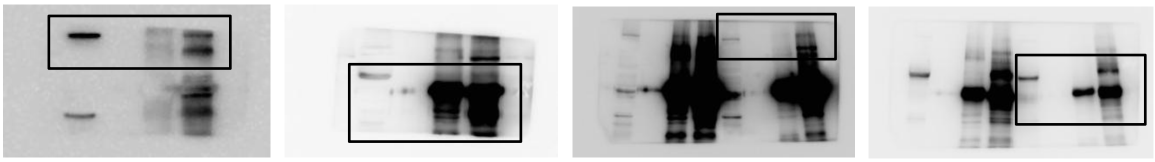

Figure 1D

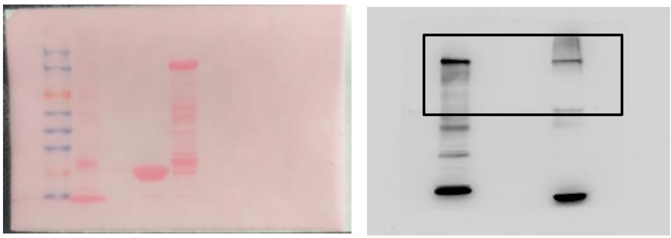

Figure 1E

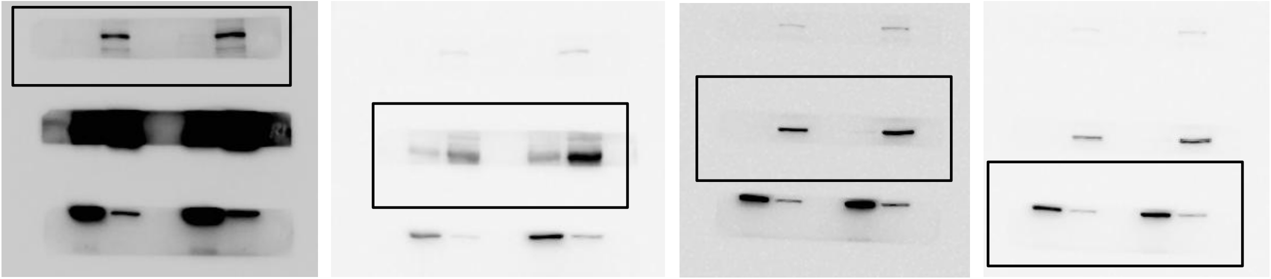

Figure 1F

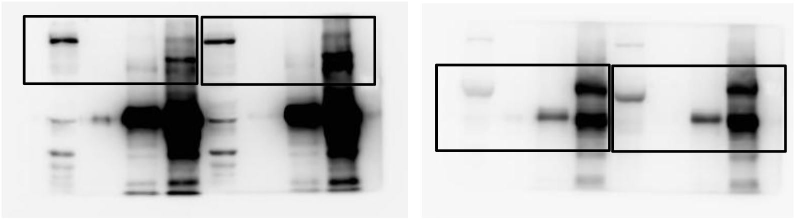

Figure 1H

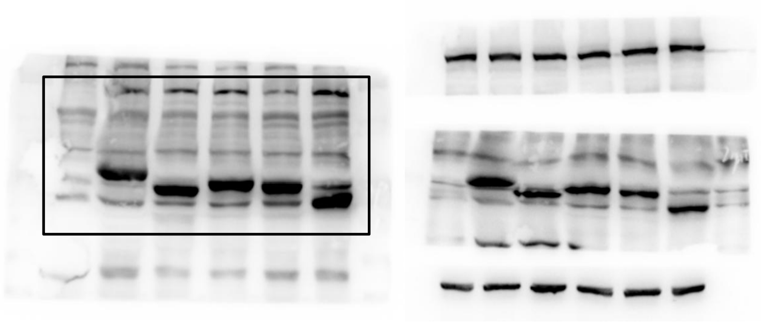

Figure 2C

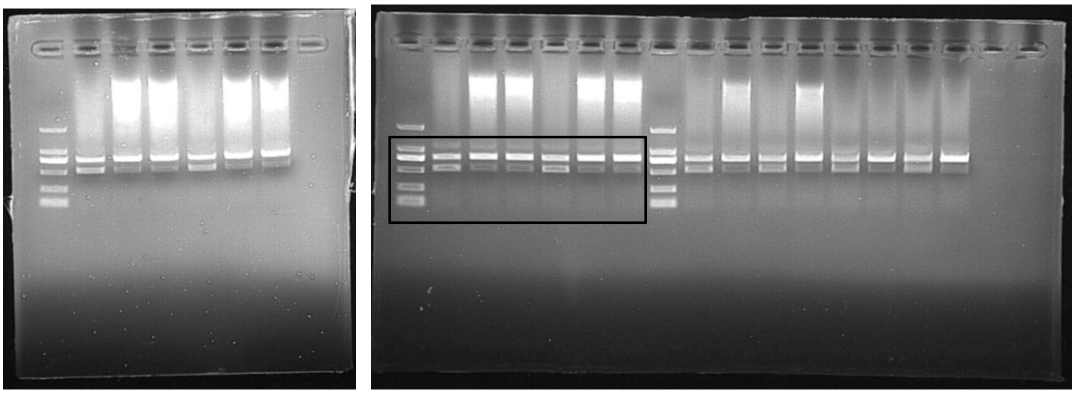

Figure 2F

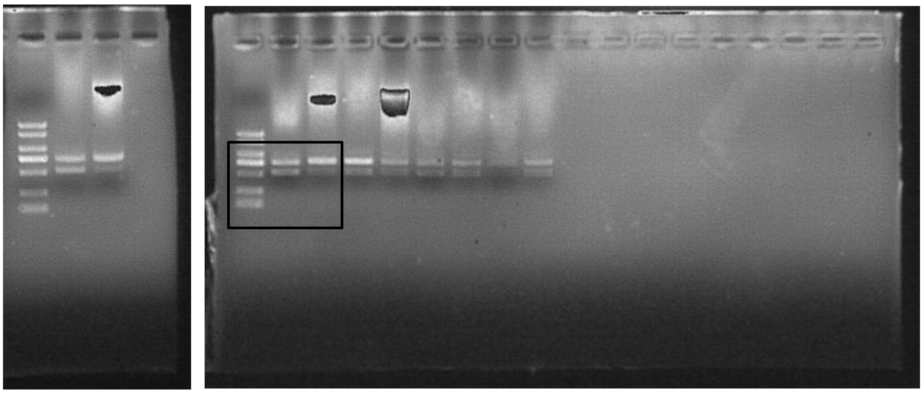

Figure 3E

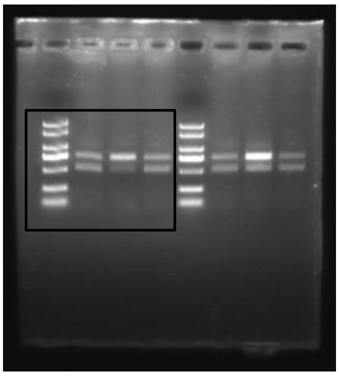

Figure 3H

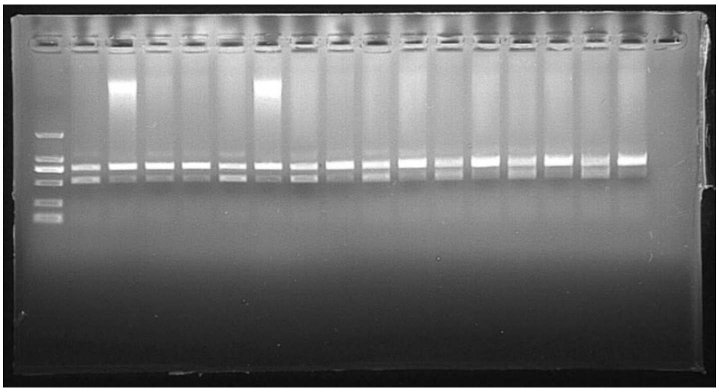

Figure 4B

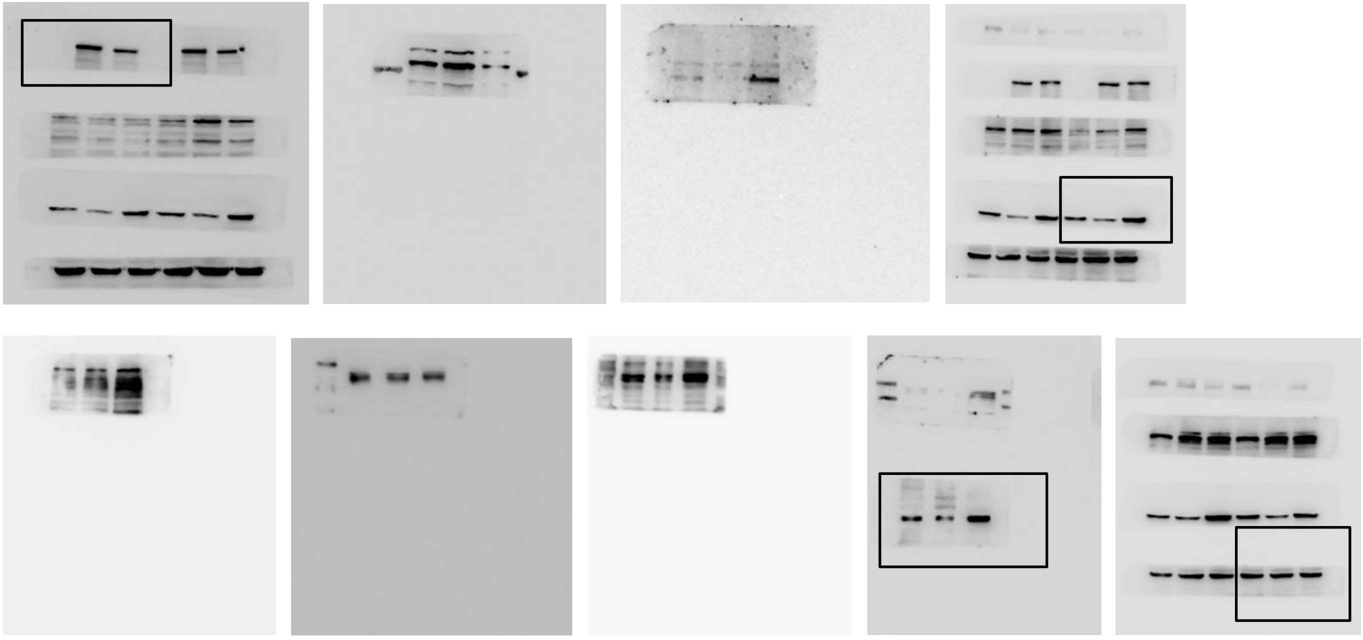

Figure 4H

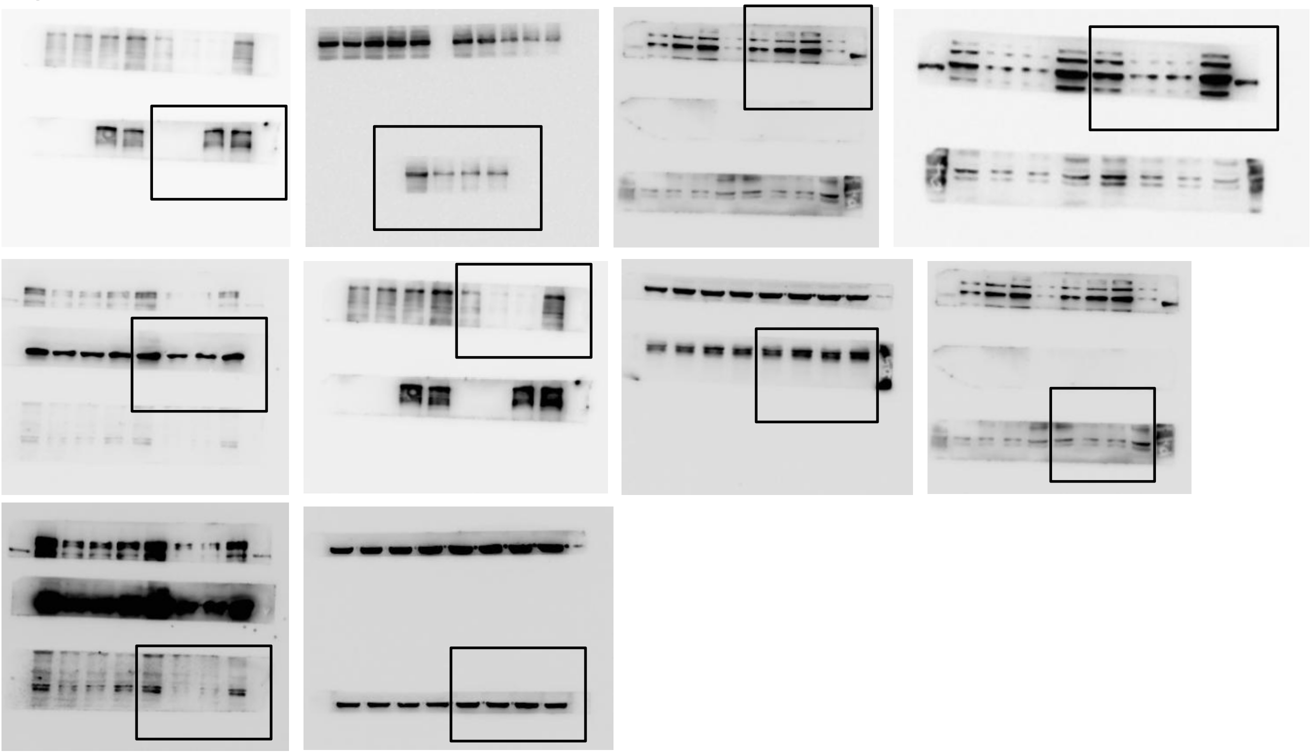

Figure 4I

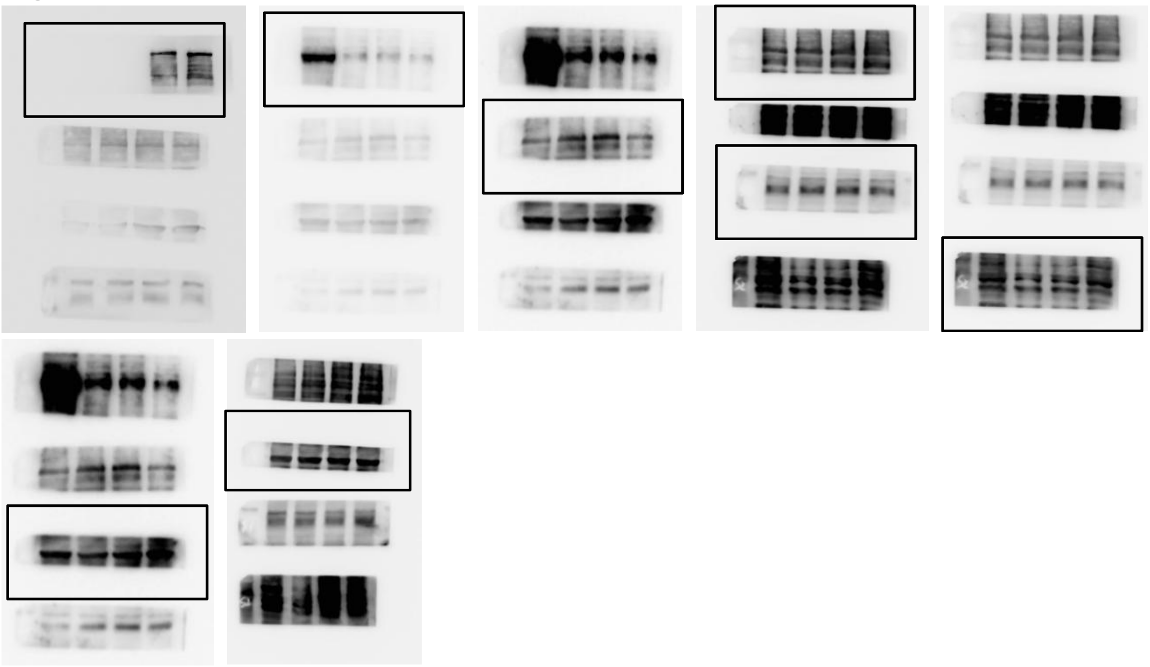

Figure 4J

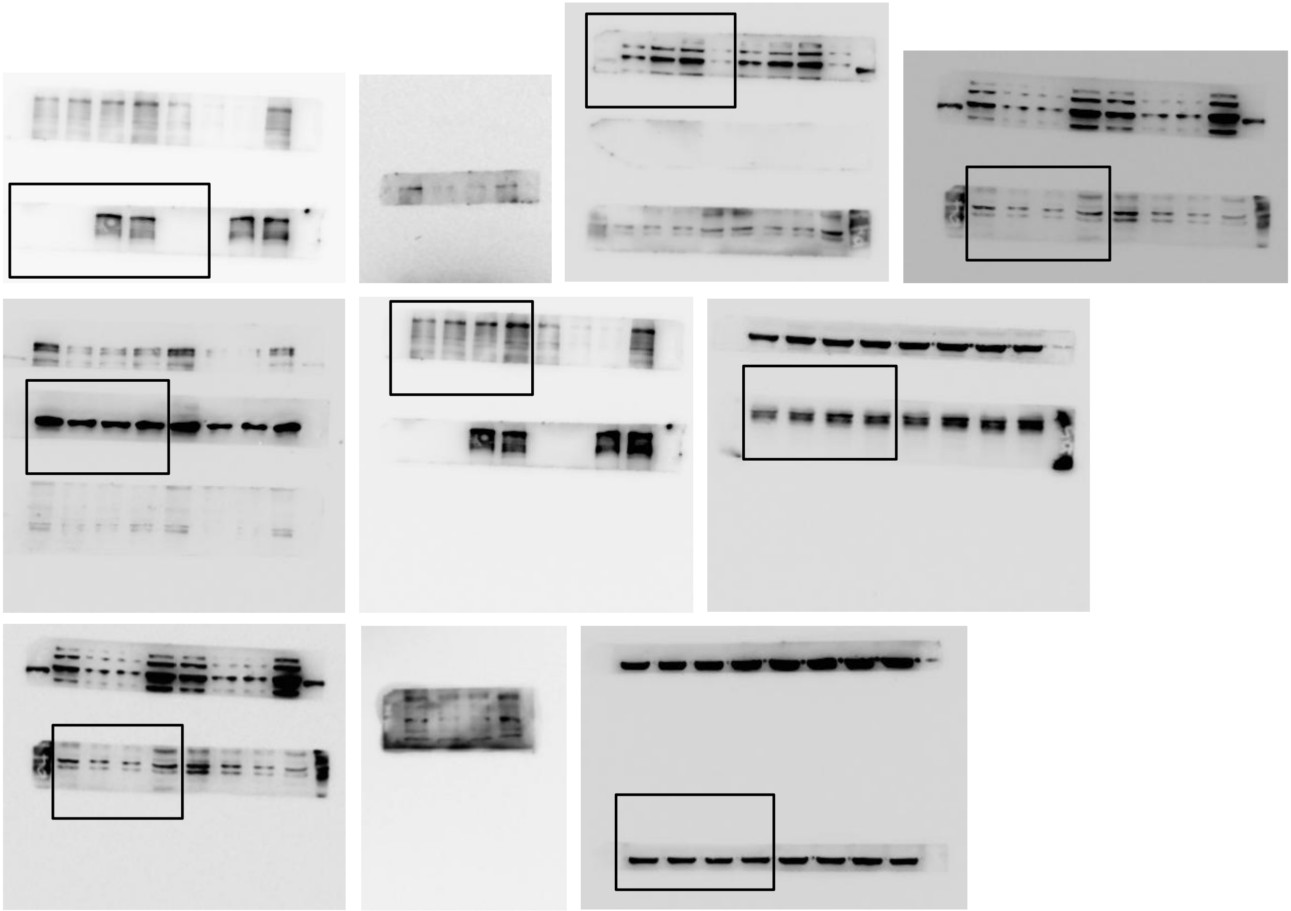

Figure 5C

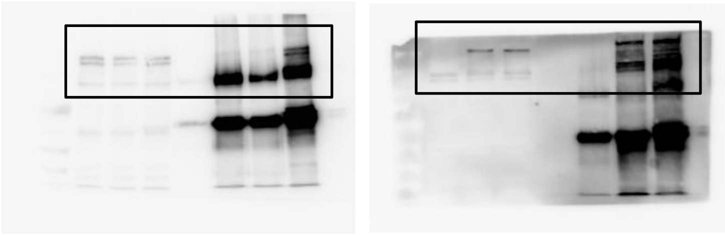

Figure 5H

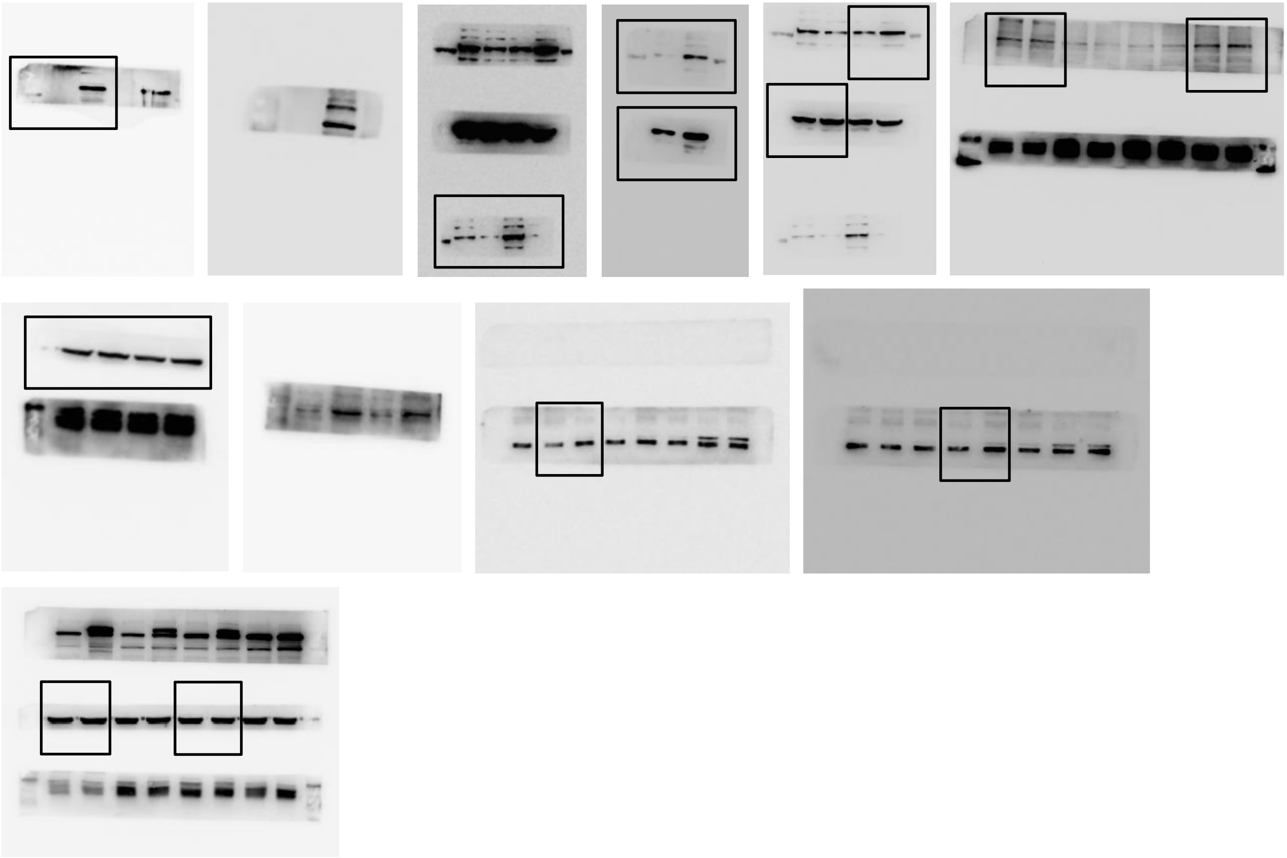

Figure 5I

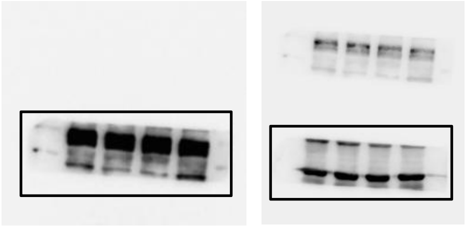

Figure 5J

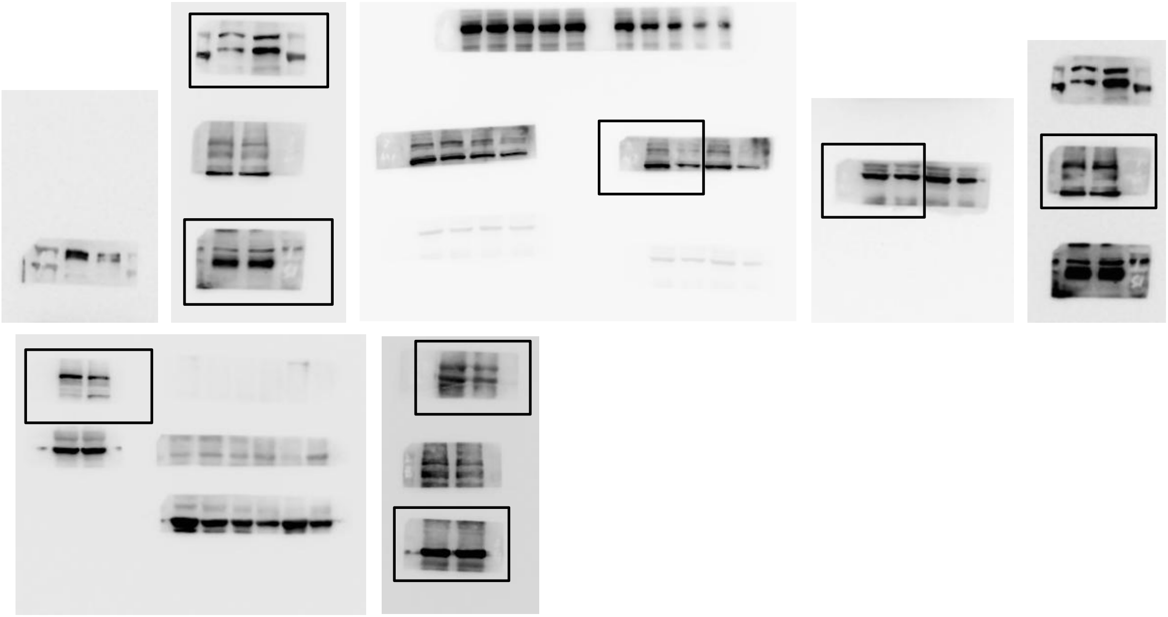

Figure 5K

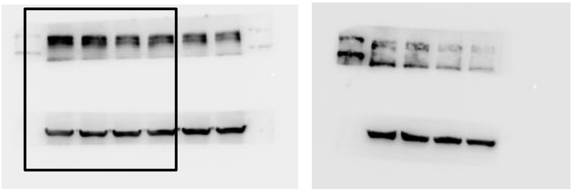

Figure 5L

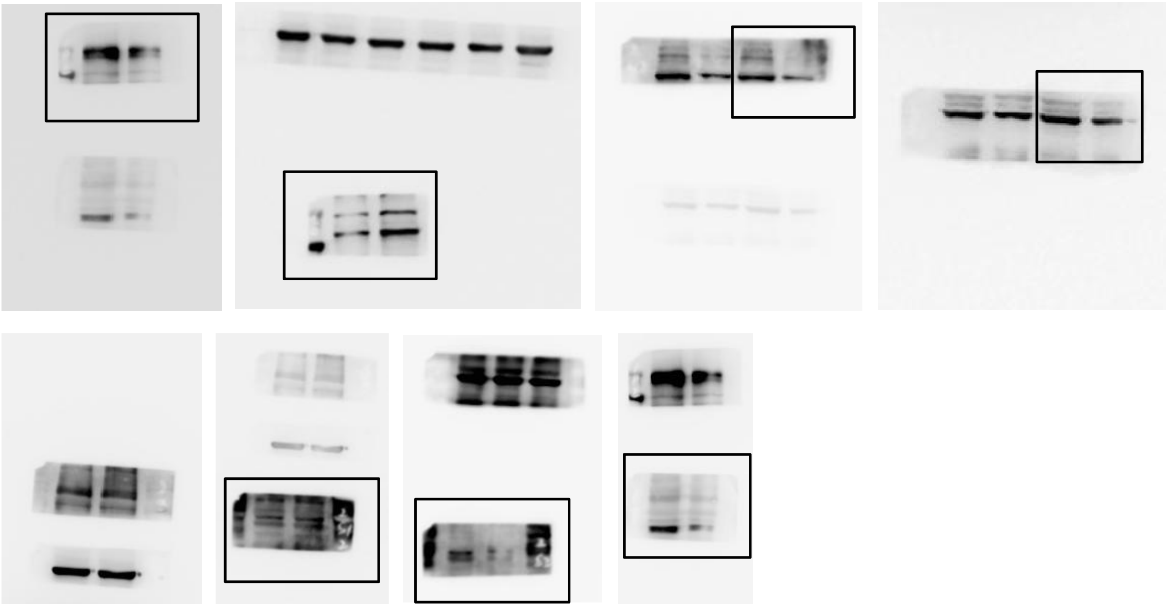

Figure 7B

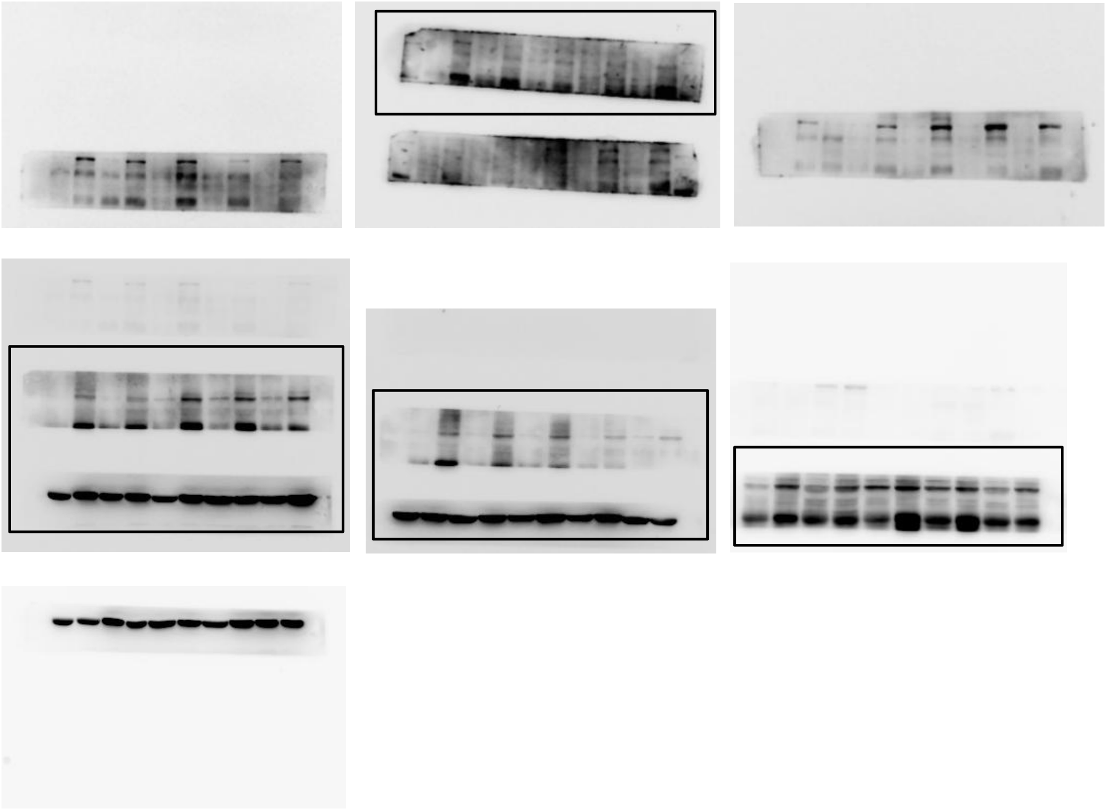

Figure 7C

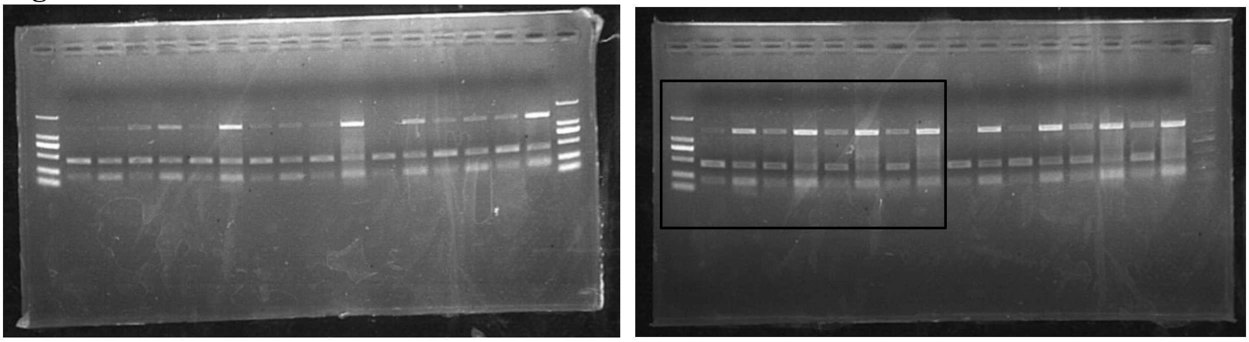

Supplementary Figure 1D

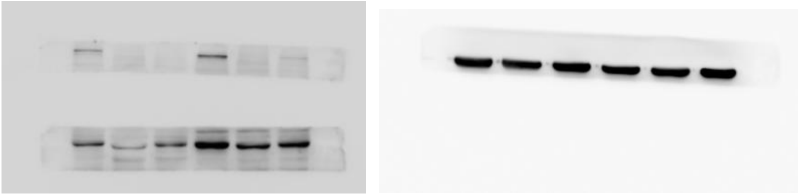

Supplementary Figure 1E

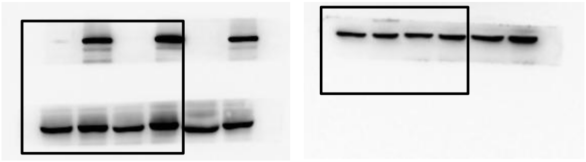

Supplementary Figure 3A

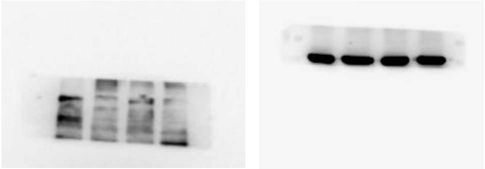

Supplementary Figure 3B

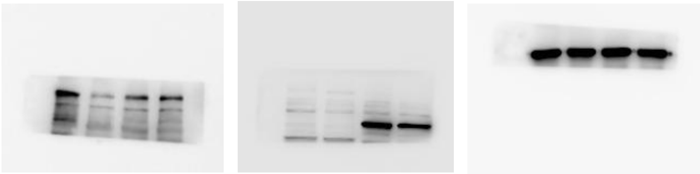

Supplementary Figure 3C

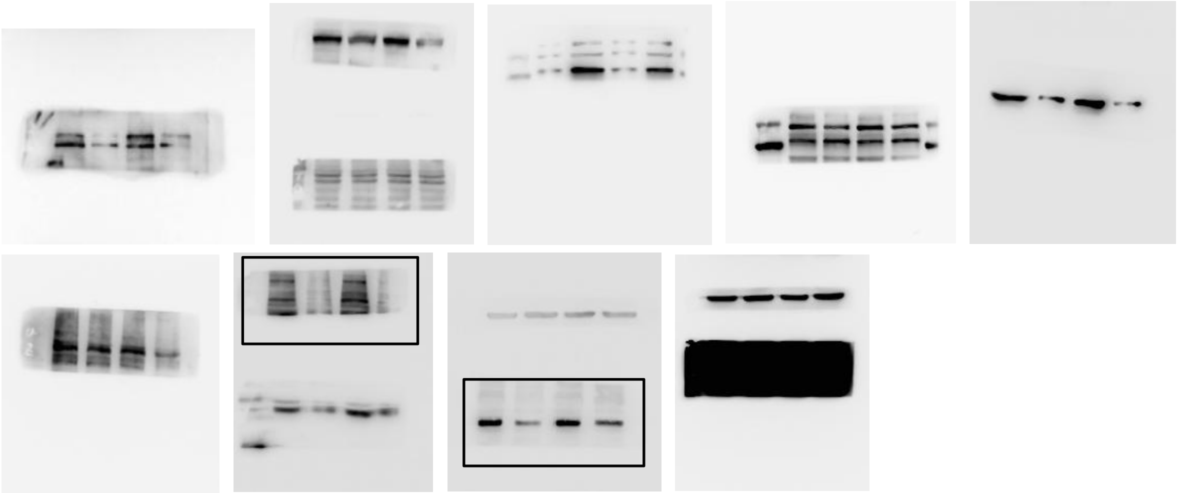

Supplementary Figure 3D

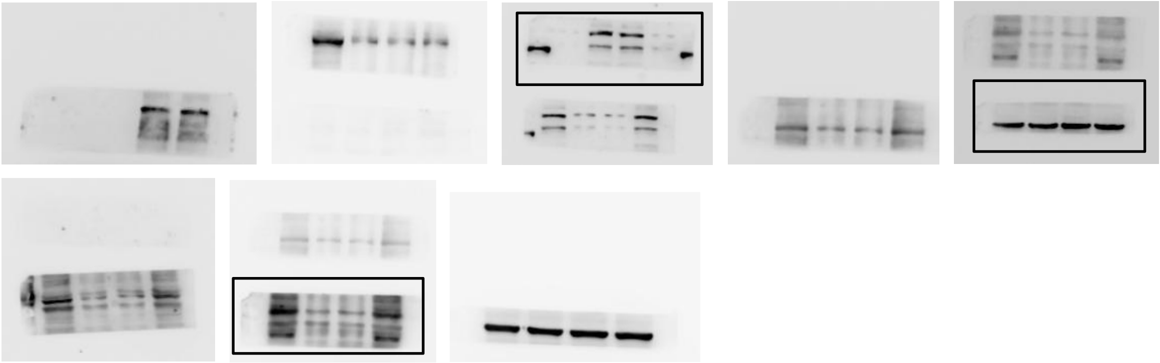

Supplement: Supplementary file 2 — Original Data File [file 41419_2024_6908_MOESM2_ESM.pdf]
